# Integrated Analysis to Identify a Redox-Related Prognostic Signature for Clear Cell Renal Cell Carcinoma

**DOI:** 10.1155/2021/6648093

**Published:** 2021-04-21

**Authors:** Yue Wu, Xian Wei, Huan Feng, Bintao Hu, Bo Liu, Yang Luan, Yajun Ruan, Xiaming Liu, Zhuo Liu, Jihong Liu, Tao Wang

**Affiliations:** ^1^Department of Urology, Tongji Hospital, Tongji Medical College, Huazhong University of Science and Technology, Wuhan, 430030 Hubei, China; ^2^Institute of Urology, Tongji Hospital, Tongji Medical College, Huazhong University of Science and Technology, Wuhan, 430030 Hubei, China; ^3^Department of Oncology, Tongji Hospital, Tongji Medical College, Huazhong University of Science and Technology, Wuhan, 430030 Hubei, China

## Abstract

The imbalance of the redox system has been shown to be closely related to the occurrence and progression of many cancers. However, the biological function and clinical significance of redox-related genes (RRGs) in clear cell renal cell carcinoma (ccRCC) are unclear. In our current study, we downloaded transcriptome data from The Cancer Genome Atlas (TCGA) database of ccRCC patients and identified the differential expression of RRGs in tumor and normal kidney tissues. Then, we identified a total of 344 differentially expressed RRGs, including 234 upregulated and 110 downregulated RRGs. Fourteen prognosis-related RRGs (*ADAM8*, *CGN*, *EIF4EBP1*, *FOXM1*, *G6PC*, *HAMP*, *HTR2C*, *ITIH4*, *LTB4R*, *MMP3*, *PLG*, *PRKCG*, *SAA1*, and *VWF*) were selected out, and a prognosis-related signature was constructed based on these RRGs. Survival analysis showed that overall survival was lower in the high-risk group than in the low-risk group. The area under the receiver operating characteristic curve of the risk score signature was 0.728 at three years and 0.759 at five years in the TCGA cohort and 0.804 at three years and 0.829 at five years in the E-MTAB-1980 cohort, showing good predictive performance. In addition, we explored the regulatory relationships of these RRGs with upstream miRNA, their biological functions and molecular mechanisms, and their relationship with immune cell infiltration. We also established a nomogram based on these prognostic RRGs and performed internal and external validation in the TCGA and E-MTAB-1980 cohorts, respectively, showing an accurate prediction of ccRCC prognosis. Moreover, a stratified analysis showed a significant correlation between the prognostic signature and ccRCC progression.

## 1. Introduction

Renal cell carcinoma (RCC) is one of the most common urogenital tumors, among which clear cell RCC (ccRCC) is the most common subtype, accounting for about 75% of all renal tumors [1]. The standard treatment for ccRCC is surgery, with a high cure rate for localized disease, early and a 5-year survival rate of more than 90%, while the 5-year survival rate for patients with distant metastases drops to 12% [2]. However, nearly 25-30% of ccRCC patients are diagnosed with advanced cancer, and 30% have distant metastases after surgery for early cancer [3, 4]. In addition, the TNM staging system (tumor, lymph node, and metastasis) currently used clinically cannot effectively predict the invasiveness of ccRCC [5]. Although some renal carcinoma-related biomarkers have been released recently, such as Li et al. [6] have developed a classification system of ccRCC based on PKM alternative splicing; Caliskan et al. [7] conducted comparative analysis of RNA-seq transcriptome data of different RCC subtypes and found reporter molecules that were specific to each other or subtype; there are still few markers or models that can be used to predict the prognosis of ccRCC patients clinically. Therefore, in-depth exploration of the molecular mechanism of ccRCC, identification of biomarkers that can effectively predict the prognosis and progression of ccRCC, and development of effective early screening and diagnosis methods are of vital importance for improving the treatment effect and quality of life of patients.

The homeostasis system of cellular redox forms a delicate balance between the production of reactive oxygen species (ROS) and the removal of reactive oxygen species by antioxidant enzymes and small-molecule antioxidants, and participates in the regulation of physiological events such as cell signal transduction, proliferation, and differentiation at normal low concentrations [8, 9]. However, excessive intracellular ROS accumulation can cause oxidative stress, which can damage cell membranes, promote mitochondrial damage, and induce cell death, thus negatively affecting cell function and survival [10–12]. It is worth noting that this is largely due to the uncontrolled increase of ROS, which leads to the accumulation of large amounts of free radicals, thus destroying proteins, DNA, and lipid macromolecules, leading to genomic instability and changes in cell growth [13]. It is therefore not surprising that disorders of redox homeostasis are associated with the development of a variety of pathologies, including obesity, diabetes, cardiovascular disease, and neurodegenerative diseases [14–16]. In the past few decades, many studies have also shown that the imbalance of the oxidation-reduction system and the accumulation of ROS and oxidative stress can mediate the occurrence and development of cancer by causing molecular damage [17, 18]. Redox imbalance was also found in the development and progression of renal cell carcinoma [19, 20]. However, there has been no systematic study on the composition of redox-related genes (RRGs) in ccRCC and their relationship with prognosis. Thus, understanding the molecular composition of RRGs and their roles and functions in ccRCC is necessary for improving prognosis and identifying new biomarkers.

In the current study, we download the transcriptome data and corresponding clinical data of ccRCC from The Cancer Genome Atlas (TCGA) database. We identified differentially expressed RRGs and found that these genes were closely related to clinical parameters. Subsequently, we identified the fourteen RRGs most associated with prognosis and constructed a predictive model based on them. Kaplan-Meier survival analysis and time-dependent receiver operating characteristic (ROC) analysis showed that the model had satisfactory predictive potential. Next, we explored the upstream regulatory network of these RRGs and its relationship with immune cell infiltration. We then built a nomogram based on the signature and other clinical parameters and validated it in the TCGA database and ArrayExpress database. Finally, we verified the expression of these RRGs in the Human Protein Atlas (HPA) database.

## 2. Materials and Methods

### 2.1. Data Access, Collation, and Differential Expression Analysis

The miRNA sequencing dataset, RNA sequencing dataset, and corresponding clinical data of ccRCC were downloaded from the TCGA (https://portal.gdc.cancer.gov/) database. Then, genes related to redox were screened from the OMIM database (https://www.oncomine.org/resource/), NCBI gene function module (https://www.ncbi.nlm.nih.gov/gene/), GeneCards database (https://www.genecards.org/), and GSEA-MSigDB (https://www.gsea-msigdb.org/gsea/msigdb) with the keyword “redox” [21]; a total of 4087 RRGs were obtained. In addition, we downloaded the E-MTAB-1980 dataset from the ArrayExpress database (https://www.ebi.ac.uk/arrayexpress/) as an external validation cohort. Next, we used edgeR package (http://www.bioconductor.org/packages/release/bioc/html/edgeR.html) to preprocess the raw data of the TCGA cohort, including averaging the genes with the same name, removing the genes with an average expression of less than 1, and normalizing the expression data based on trimmed mean of *M*-values (TMM) algorithm. And for microarray data from ArrayExpress, the data were background adjusted and normalized using the robust multiarray analysis (RMA) method in affy package (http://www.bioconductor.org/packages/release/bioc/html/affy.html). Additionally, the data were transformed using a log_2_ transformation, and the probes were converted into gene symbols. When a gene was recorded by multiple probes, its expression level was averaged. ∣Log_2_ fold change (FC) | >2.0 and false discovery rate (FDR) < 0.05 were considered to be differently expressed genes.

### 2.2. Evaluation of Gene Modules and Their Correlation with Clinical Parameters

We performed weighted correlation network analysis (WGCNA) of differentially expressed RRGs to establish gene interaction modules and to evaluate the relationships between these RRGs and clinical parameters as a whole, according to the WGCNA package. Briefly, after soft threshold (power) was set and cluster modules and genes were obtained, correlation analysis was conducted between clinical parameters (including age, gender, tumor grade, tumor stage, T stage, N stage, and M stage) and module characteristic genes. A *p* < 0.05 was considered statistically significant.

### 2.3. Establishing Protein-Protein Interaction (PPI) Network and Screening Key Modules

We first identified the protein-protein interaction information of these differentially expressed RRGs through the STRING database (http://www.string-db.org/). Then, the PPI network was constructed and visualized using Cytoscape 3.8.0 software. In addition, we used the Molecular Complex Detection (MCODE) plug-in to filter the key modules with nodes greater than 10.

### 2.4. Identification of Prognosis-Related RRGs

First, univariate Cox regression analysis was performed on these key RRGs of the TCGA cohort to identify the RRGs associated with prognosis. Subsequently, we performed the least absolute shrinkage and selection operator (LASSO) regression analysis, Kaplan-Meier test, and multivariate Cox regression analysis to screen for the RRGs most associated with prognosis. A *p* < 0.05 was considered significant.

### 2.5. Construction and Evaluation of RRG-Based Prognosis-Related Signature

After screening these prognosis-related RRGs, a multivariate Cox proportional hazards regression model was constructed to predict the prognosis of ccRCC patients. The risk score for each patient in the signature was calculated according to the following formula:

(1) $$\begin{array}{c} \text{Risk score}=\overset{n}{\underset{i=1}{\sum }}\text{E}\text{xp}i\beta i. \end{array}$$

Here, Exp represents the expression of each gene, and *β* represents the regression coefficient. Subsequently, based on the median risk score, we divided the TCGA cohort into high-risk and low-risk subgroups. Then, we performed the Kaplan-Meier survival analysis to compare the difference in overall survival (OS) between the two subgroups. And the time-dependent ROC curve was used to evaluate the prognostic ability of the signature. In addition, the E-MTAB-1980 cohort was used as an external validation set to verify the stability and accuracy of the signature. Moreover, we also randomly and equally divided the TCGA cohort into two datasets, and further verified the stability and reliability of the signature based on these two datasets.

### 2.6. The Expression Differences of Signature-Based Risk Score and Prognosis-Related RRGs Stratified by Different Clinicopathological Parameters

We analyzed the expression differences of signature-based risk score stratified by different clinicopathological parameters to explore whether it might affect the progression of ccRCC. In addition, we analyzed the expression differences of prognosis-related RRGs stratified by different clinicopathological parameters to understand the role of redox in ccRCC. A *p* < 0.05 was considered significant.

### 2.7. Upstream Regulatory Network and Functional Enrichment Analysis of Prognosis-Related RRGs

We first obtained ccRCC miRNA sequencing dataset from the TCGA database. Next, we conducted coexpression analysis of differentially expressed miRNAs and prognosis-related RRGs to explore their regulatory relationships, based on ∣Cor | >0.1 and *p* < 0.001 standard. Subsequently, the functional enrichment analysis of these differentially expressed RRGs was detected by the Gene Ontology (GO) and Kyoto Encyclopedia of Genes and Genomes (KEGG) database pathway enrichment analysis. All enrichment analyses were performed by using the clusterProfiler package (http://www.bioconductor.org/packages/release/bioc/html/clusterProfiler.html).

### 2.8. The Infiltration Difference of Tumor-Infiltrating Immune Cells between High-Risk and Low-Risk Groups in the TCGA Cohort Assessed by RRG-Based Prognostic Signature

The degree of infiltration of immune cells in the immune microenvironment is important for tumor progression, treatment, and prognosis. We used the cell-type identification by estimating relative subsets of RNA transcripts (CIBERSORT) and its supplied LM22 gene set to assess the degree of immune cell infiltration in different subgroups. CIBERSORT is a deconvolution algorithm that assesses the relative abundance of immune cell infiltration in each patient based on the expression data of 22 tumor-infiltrating lymphocyte subsets. Here, the number of permutations was set to 1000. *p* < 0.05 was the filtering criterion.

### 2.9. Construction of a Nomogram

We performed the Cox regression analysis and multiple regression analysis to assess the prognostic significance of different clinical parameters and the prognosis-related signature. Then, to establish a quantitative approach to predict the prognosis of ccRCC patients, we constructed a nomogram combining clinical parameters and RRG-based prognosis-related signature by using rms package. Subsequently, calibration curves at different time intersections were plotted to assess the predictive accuracy of the nomograms. And the TCGA and E-MTAB-1980 datasets were used for Kaplan-Meier survival analysis and ROC analysis to further evaluate the accuracy and stability of the nomogram.

### 2.10. Validation of Prognosis-Related RRG Expression

We used the immunohistochemical results from the Human Protein Atlas (HPA, http://www.proteinatlas.org/) online database to detect the protein expression of these prognosis-related RRGs [22].

## 3. Results

### 3.1. Identifying Differentially Expressed RRGs

**Figure 1:**
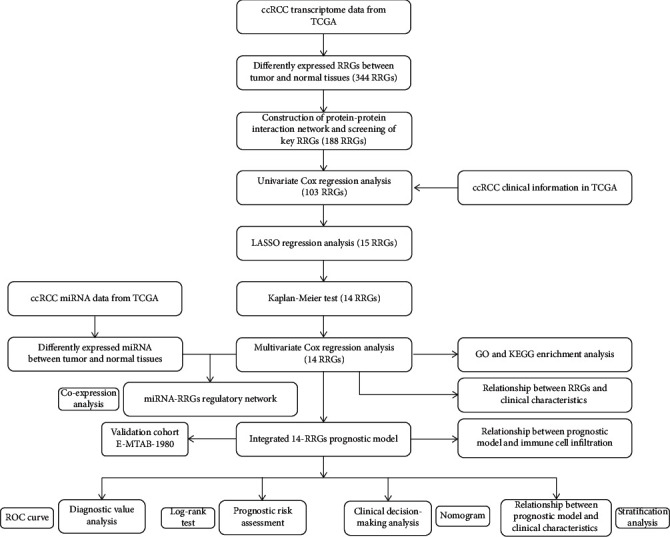
The flow chart for analyzing RRG-based model and miRNA-RRG regulatory network in ccRCC.

**Figure 2:**
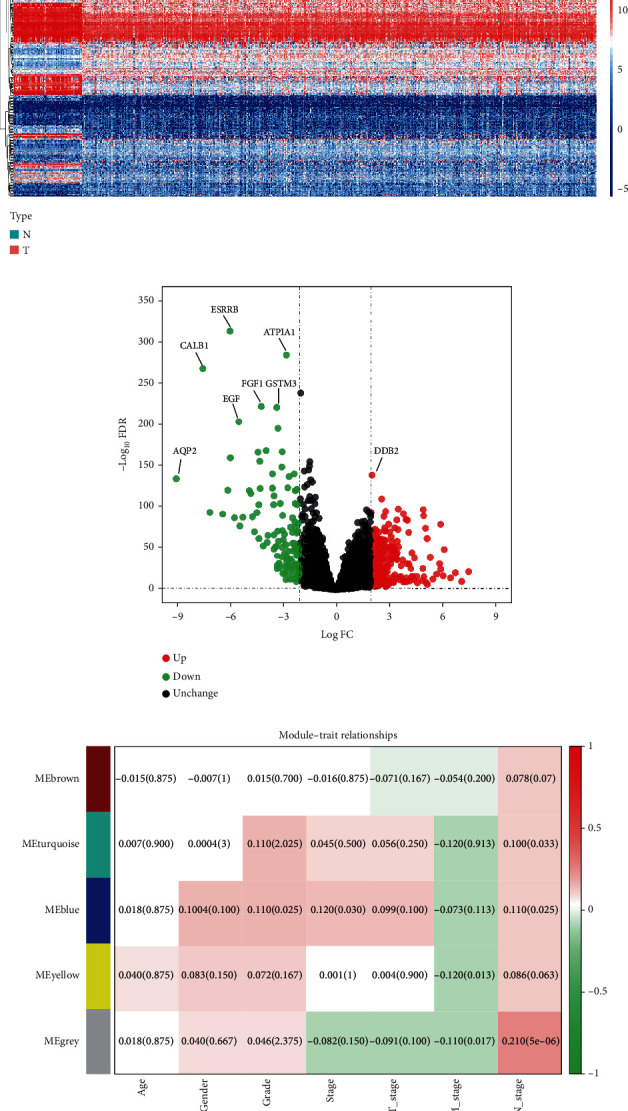
Landscape of the expression and distribution of differentially expressed RRGs in ccRCC and the correlation between gene module and clinical parameters based on WGCNA analysis. (a) Heat map of 344 differentially expressed RRGs in the normal renal tissues and ccRCC tissues. (b) Volcano plot shows the log_2_ fold change and *q* value of each differentially expressed RRG. (c) Module-trait relationships based on WGCNA analysis. Each column represents a clinical trait and each row represents a gene module.

In this study, we systematically and comprehensively analyzed the role and clinical significance of RRGs in ccRCC.  Figure 1 shows a flow chart of the study. A total of 72 normal renal tissue samples and 539 ccRCC samples were analyzed. We identified a total of 4087 RRGs from the GeneCards, OMIM, NCBI, and GSEA-MSigDB databases, and finally, 3845 RRG expression data was obtained according to the TCGA cohort. Next, based on our inclusion criteria (∣log_2_ FC | >2.0 and FDR < 0.05), 344 differentially expressed RRGs were identified, including 234 upregulated and 110 downregulated RRGs. The expression distribution of these RRGs is shown in Figures 2(a) and 2(b).

### 3.2. Correlation between Gene Modules and Clinical Characteristics

We performed WGCNA analysis to determine the correlation between gene modules and clinical features. Briefly, after extracting gene expression data and corresponding clinical data from the TCGA database, including prognosis status, age, gender, tumor grade, tumor stage, T stage, N stage, and M stage, we then set a soft threshold (power) and obtained the optimal scale-free topology fitting model index (scale-free *R*^2^) and average connectivity. The degree of difference among genes was determined based on topological overlap measure, and the clustering tree diagram of genes was obtained. Finally, the clinical factors and module characteristic genes in TCGA were analyzed by cluster analysis.  Figure 2(c) shows the relationships between different gene modules and clinical features such as age, gender, tumor grade, tumor stage, T stage, N stage, and M stage after WGCNA analysis. Two modules were significantly correlated with tumor grade (*p* = 0.025, *p* = 0.025). One module was significantly correlated with tumor stage (*p* = 0.030). Three modules were negatively correlated with M stage (*p* = 0.013, *p* = 0.013, and *p* = 0.017). Three modules were significantly correlated with N stage (*p* = 0.033, *p* = 0.025, and *p* < 0.001). However, there was no significant correlation between the gene models and age, gender, and T stage. Although our results showed a small effect size, the association was statistically significant, suggesting that RRGs may affect clinical outcomes in ccRCC patients. Therefore, prognostic analysis deserved to be performed subsequently.

### 3.3. Construction of PPI Network and Screening Key Modules

**Figure 3:**
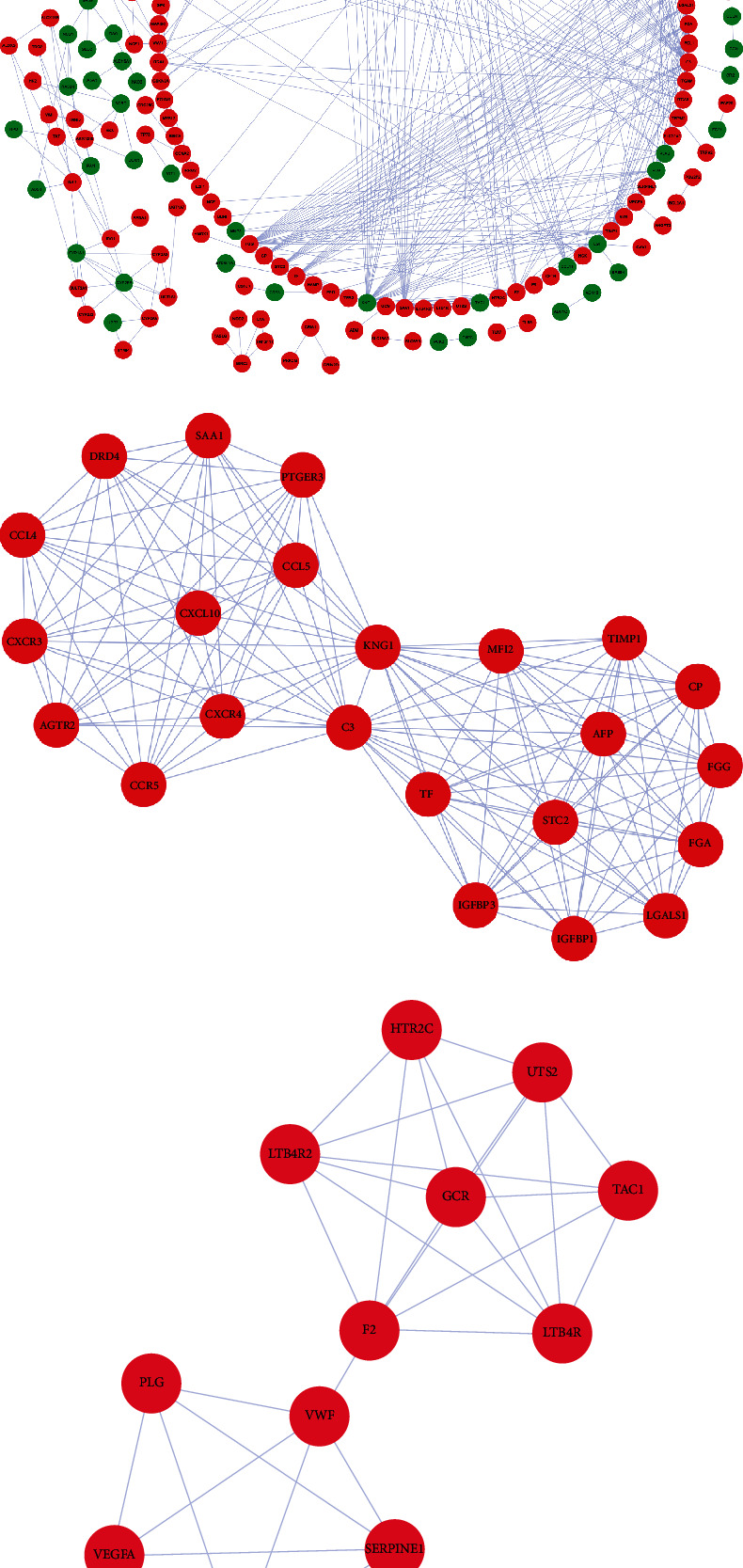
Construction of protein-protein interaction network and screening key modules. (a) Protein-protein interaction network of differentially expressed RRGs. (b) Critical module 1 from PPI network based on MCODE plug-in. (c) Critical module 2 from PPI network based on MCODE plug-in. Green circles: downregulation; red circles: upregulation.

In order to further explore the role of key RRGs in ccRCC, we used the STRING database and Cytoscape software to analyze these differentially expressed RRGs and construct a PPI network containing 189 nodes and 489 edges (Figure 3(a)). We also used the MCODE plug-in to filter two key modules. Module 1 contained 23 nodes and 143 edges (Figure 3(b)). And module 2 contained 12 nodes and 32 edges (Figure 3(c)).

### 3.4. Construction and Evaluation of RRG-Based Prognosis-Related Signature

We first performed univariate Cox regression analysis on these 189 key RRGs and identified 103 prognosis-related RRGs (Supplemental Table S2). Next, LASSO regression analysis was performed for further analysis, and 15 RRGs were identified (Supplemental Figure S1). To further identify the RRGs with the best prognostic significance, we identified 14 RRGs, including *ADAM8*, *CGN*, *EIF4EBP1*, *FOXM1*, *G6PC*, *HAMP*, *HTR2C*, *ITIH4*, *LTB4R*, *MMP3*, *PLG*, *PRKCG*, *SAA1*, and *VWF*, by using the Kaplan-Meier test (Supplemental Figure S2). Next, the GEPIA online tool (http://gepia.cancer-pku.cn/) was used to explore the expression levels of these 14 RRGs in different cancer types in the TCGA cohort, and the results are shown in Supplemental Figure S3. Subsequently, a RRG-based prognosis-related signature was established by multiple stepwise Cox regression (Table 1). The risk score of each ccRCC patient was calculated as follows:

(2) $$\begin{array}{c} \text{Risk score}=\left(0.0632\times \text{Exp ADAM}8\text{ reads}\right)+\left(-0.0989\times \text{Exp CGN reads}\right)+\left(0.1336\times \text{Exp EIF}4\text{EBP}1\text{ reads}\right)+\left(0.1039\times \text{Exp FOXM}1\text{ reads}\right)+\left(-0.0263\times \text{Exp G}6\text{PC reads}\right)+\left(0.0258\times \text{Exp HAMP reads}\right)+\left(0.1703\times \text{Exp HTR}2\text{C reads}\right)+\left(0.0460\times \text{Exp ITIH}4\text{ reads}\right)+\left(0.1244\times \text{Exp LTB}4\text{R reads}\right)+\left(0.0618\times \text{Exp MMP}3\text{ reads}\right)+\left(-0.0531\times \text{Exp PLG reads}\right)+\left(0.0259\times \text{Exp PRKCG reads}\right)+\left(0.0332\times \text{Exp SAA}1\text{ reads}\right)+\left(-0.0657\times \text{Exp VWF reads}\right). \end{array}$$

**Figure 4:**
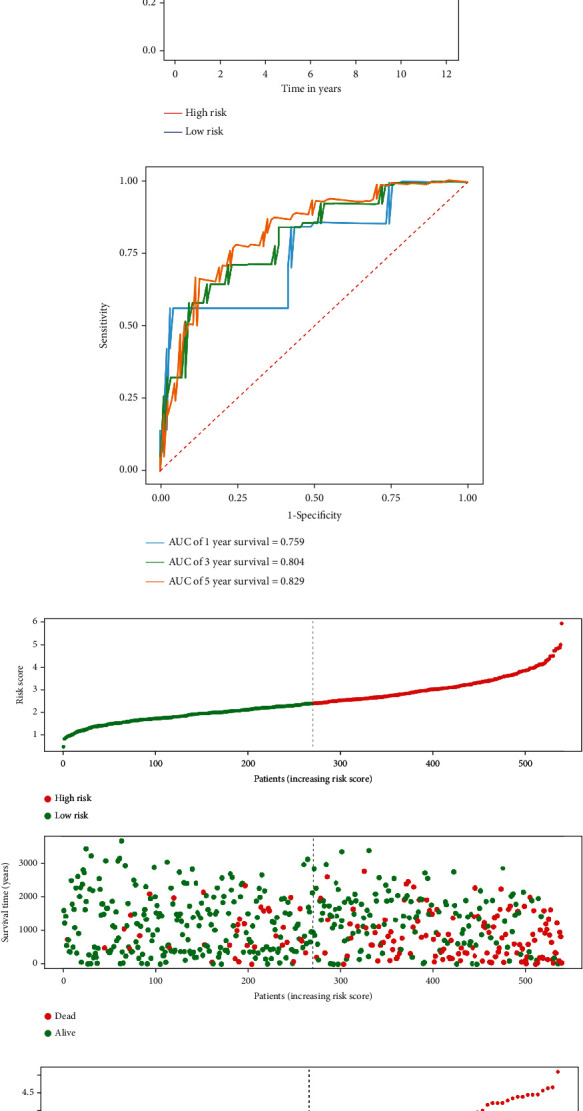
Risk score, survival time, and survival status analysis of ccRCC patients based on the fourteen RRGs' prognostic signature in the TCGA and E-MTAB-1980 cohorts. (a) Kaplan-Meier survival curve analysis of OS in the high- and low-risk subgroups of the TCGA cohort. ccRCC patients were grouped according to the median risk score. (b) Time-dependent ROC curves of the RRG-based risk signature for the TCGA cohort. The ROC curves and AUC were shown to predict ccRCC patients at 1, 3, and 5 years. (c) Kaplan-Meier survival curve analysis of OS in the high- and low-risk subgroups of the E-MTAB-1980 cohort. ccRCC patients were grouped according to the median risk score. (d) Time-dependent ROC curves of the RRG-based risk signature for the E-MTAB-1980 cohort. The ROC curves and AUC were shown to predict ccRCC patients at 1, 3, and 5 years. (e) The survival status of each patient in the TCGA cohort assessed by risk score. (f) The survival status of each patient in the E-MTAB-1980 cohort assessed by risk score. (g) Heat map of the fourteen RRGs in the TCGA cohort was evaluated based on risk score combined with other clinical parameters. (h) Heat map of the fourteen RRGs in the E-MTAB-1980 cohort was evaluated based on risk score combined with other clinical parameters.

**Figure 5:**
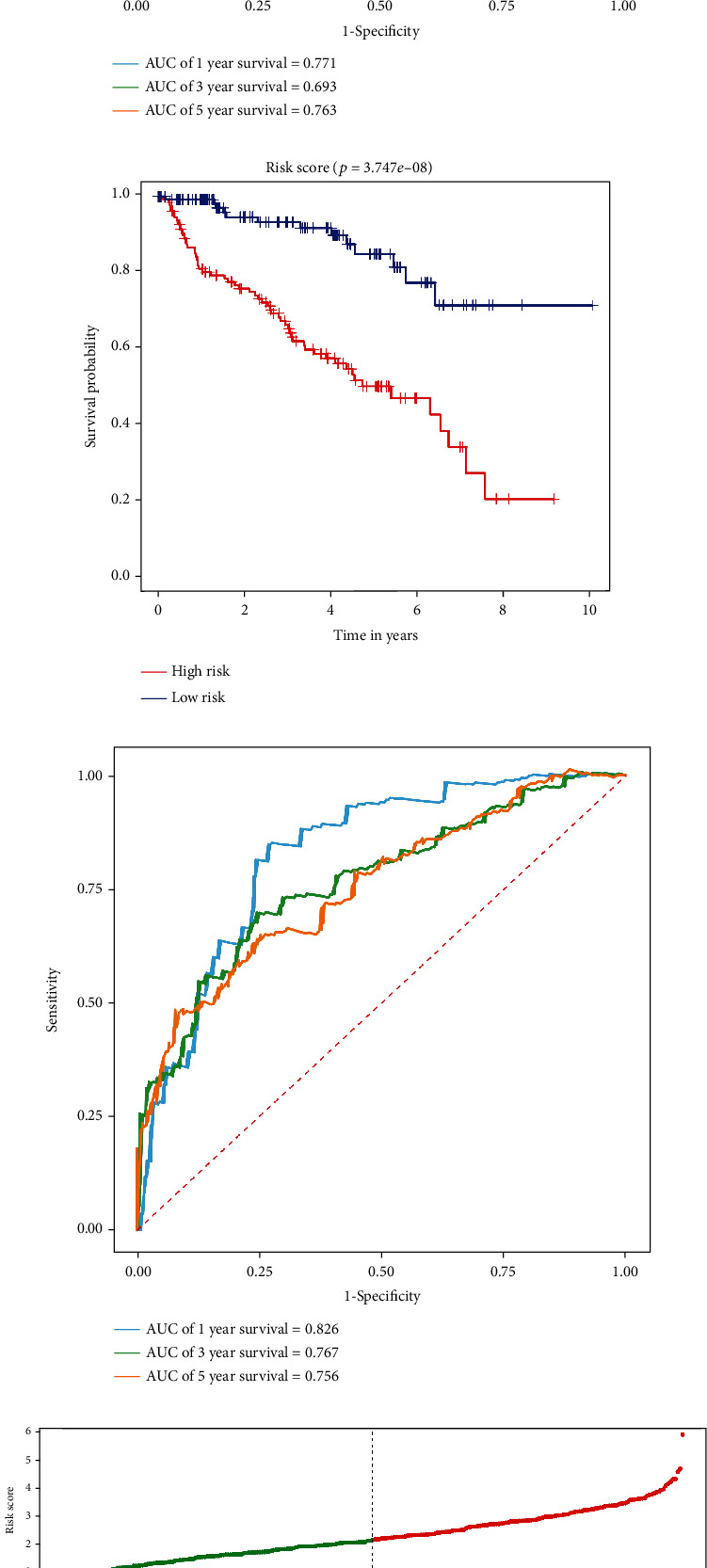
Risk score, survival time, and survival status analysis of ccRCC patients based on the fourteen RRGs' prognostic signature in the training and test groups. (a) Kaplan-Meier survival curve analysis of OS in the high- and low-risk subgroups of the training group. ccRCC patients were grouped according to the median risk score. (b) Time-dependent ROC curves of the RRG-based risk signature for the training group. The ROC curves and AUC were shown to predict ccRCC patients at 1, 3, and 5 years. (c) The survival status of each patient in the training group assessed by risk score. (d) Kaplan-Meier survival curve analysis of OS in the high- and low-risk subgroups of the test group. ccRCC patients were grouped according to the median risk score. (e) Time-dependent ROC curves of the RRG-based risk signature for the test group. The ROC curves and AUC were shown to predict ccRCC patients at 1, 3, and 5 years. (f) The survival status of each patient in the test group assessed by risk score.

Then, according to the median risk score, the TCGA cohort was divided into high-risk and low-risk subgroups. Kaplan-Meier survival analysis showed that patients in the high-risk group had a worse prognosis than those in the low-risk group (*p* = 1.033*e* − 14, Figure 4(a)). A time-dependent ROC curve was performed to further evaluate the predictive performance of the signature, and the area under the ROC curve (AUC) for OS was 0.796 at one year, 0.728 at three years, and 0.759 at five years (Figure 4(b)). Next, the external cohort E-MTAB-1980 dataset was used to verify the stability of the RRG-based signature. The Kaplan-Meier survival analysis also showed a poorer prognosis for patients in the high-risk group (*p* = 1.164*e* − 05, Figure 4(c)). The AUCs of the 1-, 3-, and 5-year survival rates were 0.759, 0.804, and 0.829, respectively (Figure 4(d)). Figures 4(e), 4(g) and 4(f), 4(h) show the survival status and expression heat maps of each patient in the TCGA and E-MTAB-1980 cohort, respectively. Moreover, to further verify the accuracy and stability of the signature, the whole TCGA cohort was randomly divided into training (*n* = 270) and test groups (*n* = 269) for subsequent analysis. The Kaplan-Meier survival analysis also showed a worse prognosis in the high-risk group in both datasets (*p* = 1.484*e* − 08 and *p* = 3.747*e* − 08, Figures 5(a) and 5(c)). In the training dataset, the predicted AUCs for 1-, 3-, and 5-year survival rates were 0.771, 0.693, and 0.763, respectively (Figure 5(b)), and in the test dataset, the predicted AUCs for 1-, 3-, and 5-year survival rates were 0.826, 0.767, and 0.756, respectively (Figure 5(d)). Figures 5(e) and 5(h) show the survival status of each patient in the training and test groups, respectively. These results showed that the RRG-based prognosis-related signature has good predictive performance and stability.

### 3.5. Prognostic Value of the Signature Stratified by Clinical Parameters

**Figure 6:**
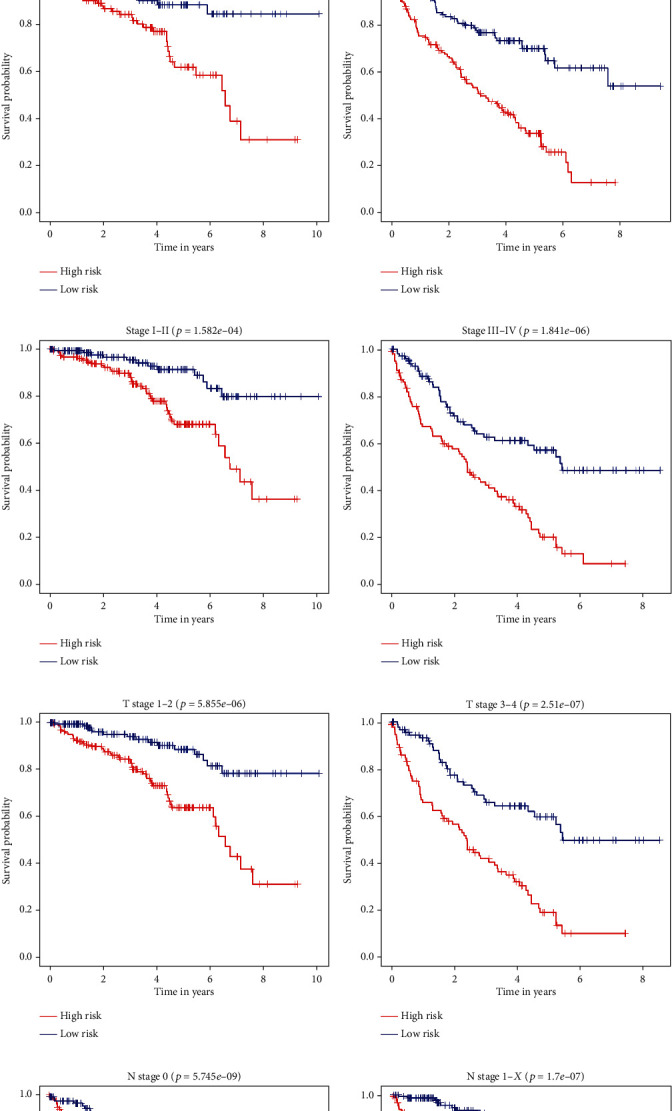
Kaplan-Meier survival analysis of ccRCC patients stratified by different clinical parameters. (a) Age ≤ 65. (b) Age > 65. (c) Male. (d) Female. (e) Grades 1-2. (f) Grades 3-4. (g) Stages I-II. (h) Stages III-IV. (i) T stages 1-2. (j) T stages 3-4. (k) N stage 0. (l) M stage 1-*X*. (m) M stage 0. (n) M stage 1-*X*.

To investigate the clinical prognostic value of the 14 RRGs-based prognosis-related signature in the ccRCC patients stratified by different clinical parameters, ccRCC patients were stratified by age, gender, tumor grade, tumor stage, T stage, N stage, and M stage. Kaplan-Meier survival analysis showed poor prognosis in all high-risk groups (Figure 6). These results suggested that the RRG-based prognosis-related signature could predict the prognosis of ccRCC patients without considering clinical parameters.

### 3.6. The Expression Differences of Signature-Based Risk Score Stratified by Different Clinicopathological Parameters

**Figure 7:**
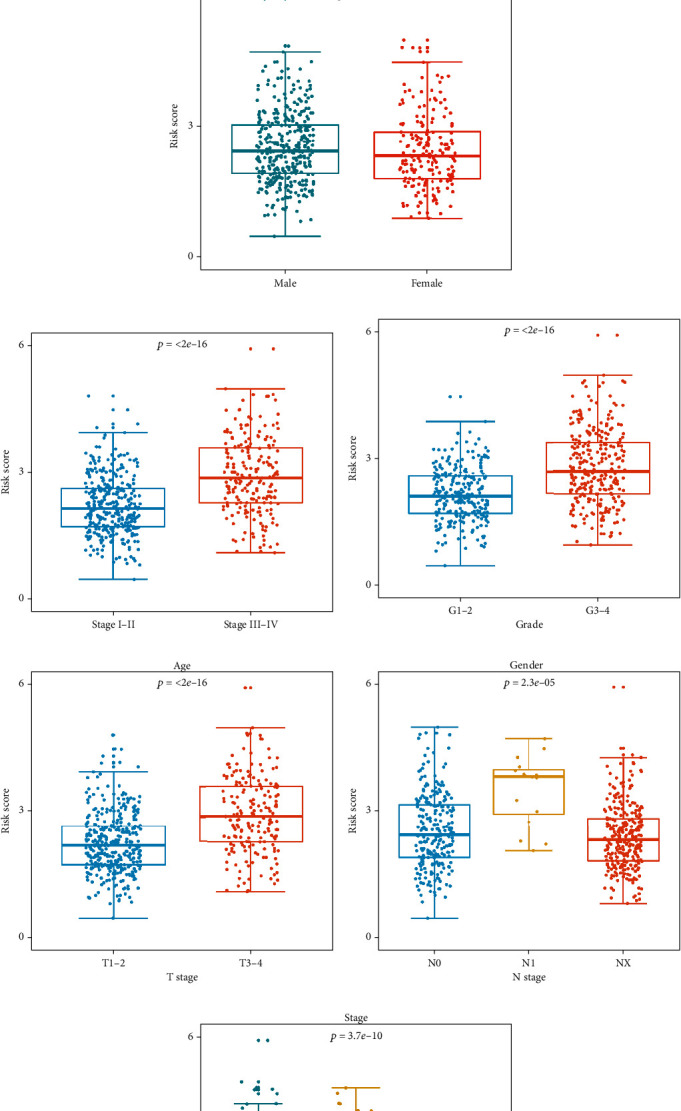
The expression differences of signature-based risk score stratified by different clinicopathological parameters. (a) Age. (b) Gender. (c) Stage. (d) Grade. (e) T stage. (f) N stage. (g) M stage.

Next, to explore whether the signature would affect the ccRCC progression, we investigated the correlation between the signature and different clinical parameters. The results showed that there was no significant correlation between age, gender, N stage, and the signature (*p* = 0.174, *p* = 0.321, and *p* = 0.281, Figures 7(a), 7(b), and 7(f)). However, the risk score of stage I-II was significantly lower than that of stage III-IV (*p* < 0.001, Figure 7(c)), the risk score of grades 1-2 was significantly lower than that of grades 3-4 (*p* < 0.001, Figure 7(d)), the risk score of T1-2 was significantly lower than that of T3-4 (*p* < 0.001, Figure 7(e)), and the risk score of M0 was significantly lower than that of M1-*X* (*p* < 0.001, Figure 7(g)). These results indicated that the prognostic signature was significantly associated with tumor progression in ccRCC, and the higher the risk score, the more advanced the tumor was.

### 3.7. The Expression Differences of Prognosis-Related RRGs Stratified by Different Clinicopathological Parameters

Based on the above results, we analyzed the relationship between prognosis-related RRGs and different clinical parameters to further investigate the role of these RRGs in ccRCC. The results showed that the expressions of G6PC and SAA1 were significantly correlated with gender; the expressions of ADAM8, CGN, EIF4EBP1, FOXM1, G6PC, HAMP, HTR2C, ITIH4, LTB4R, MMP3, PLG, PRKCG, SAA1, and VWF were significantly correlated with grade; the expressions of ADAM8, CGN, EIF4EBP1, FOXM1, G6PC, HAMP, ITIH4, LTB4R, MMP3, PLG, PRKCG, SAA1, and VWF were significantly correlated with stage and T stage; the expressions of ADAM8, EIF4EBP1, G6PC, HAMP, LTB4R, PLG, SAA1, and VWF were significantly correlated with M stage. However, no genes were associated with age and N stage (Table 2).

### 3.8. Multidimensional Regulatory Network and Functional Enrichment Analysis of Prognosis-Related RRGs

**Figure 8:**
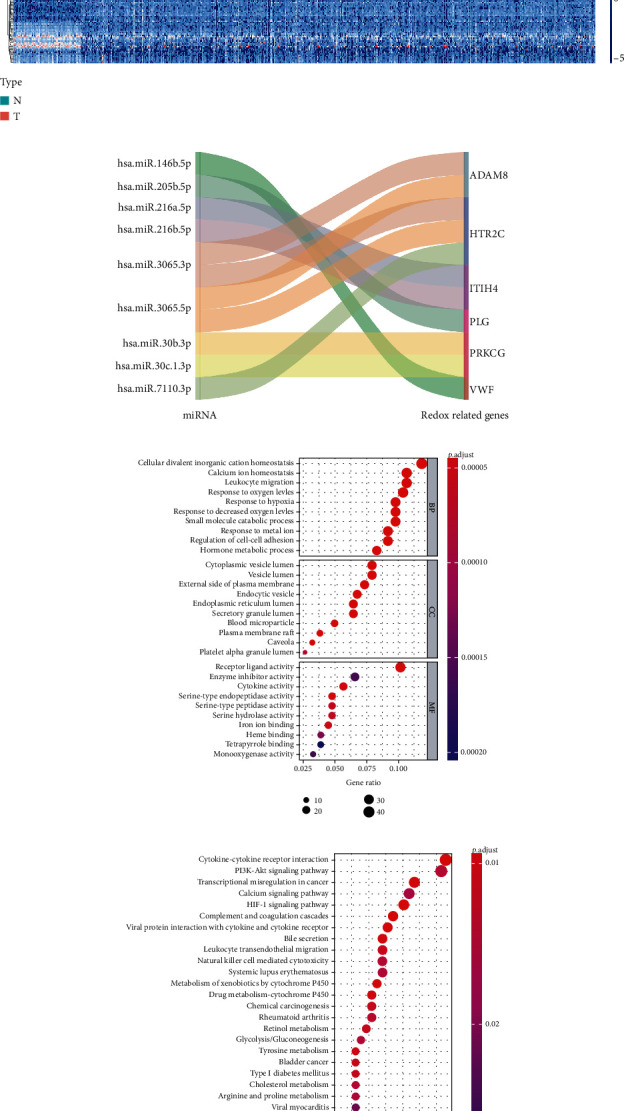
Multidimensional regulatory network of prognosis-related RRGs and differentially expressed miRNAs and the functional enrichment analysis of these RRGs. (a) Heat map of 211 differentially expressed miRNAs in the normal renal tissues and ccRCC tissues. (b) Sankey plot of the regulatory relationship between miRNAs and prognosis-related RRGs. (c) GO enrichment analysis of the differentially expressed RRGs. The top 10 enrichment analysis results, including biological processes, cell components, and molecular functions, are shown in the figure. (d) KEGG enrichment analysis of the differentially expressed RRGs. The first 30 results of functional enrichment analysis are shown in the figure.

The redox-dependent regulation of cell homeostasis is considered to be a multilayered process involving not only protein and enzyme complexes but also noncoding RNAs [23, 24]. These noncoding RNAs, including miRNAs, play important roles in regulating cellular redox homeostasis systems [25]. Some miRNAs have been found to be involved in cellular reactions by altering the expression of genes encoding antioxidant enzymes (SOD, catalase, peroxidase, and glutathione transferase) [26]. Zhang et al. [27] found that miR-206 induces ROS accumulation in vivo and in vitro by binding to SOD1 mRNA, which may be a cause of cardiovascular disease. Gómez de Cedrón et al. [28] reported that miR-661 regulates redox and metabolic homeostasis in colon cancer. Therefore, it is noteworthy to reveal the multidimensional regulatory network in tumor genesis and progression of prognosis-related RRGs and miRNAs in this study. We first investigated the upstream mechanism of RRGs based on the prognosis-related signature. We obtained 2089 miRNA sequencing data from the TCGA database, and 211 miRNAs were obtained after differential analysis, including 115 upregulated and 96 downregulated miRNAs (Figure 8(a)). Next, we conducted coexpression analysis between differentially expressed miRNAs and prognosis-related RRGs, identified a total of 9 miRNAs involved in upstream regulation, and drew a Sankey plot (Figure 8(b)). And all miRNAs positively regulated the corresponding RRGs (Supplemental Table S3).

Subsequently, we conducted GO and KEGG enrichment analysis of these RRGs by using clusterProfiler package to explore the biological functions and molecular mechanisms of these differentially expressed RRGs. GO and KEGG enrichment analysis showed that these RRGs were mainly involved in reactive oxygen species metabolic process, calcium ion homeostasis, antigen processing, treatment, peptide antigen presentation, HIF-1 signaling pathway, transcriptional misregulation in cancer, and PI3K-Akt signaling pathway (Figures 8(c) and 8(d)).

### 3.9. The Infiltration Difference of Tumor-Infiltrating Immune Cells between High-Risk and Low-Risk Groups in the TCGA Cohort Assessed by Fourteen RRG-Based Prognostic Signature

**Figure 9:**
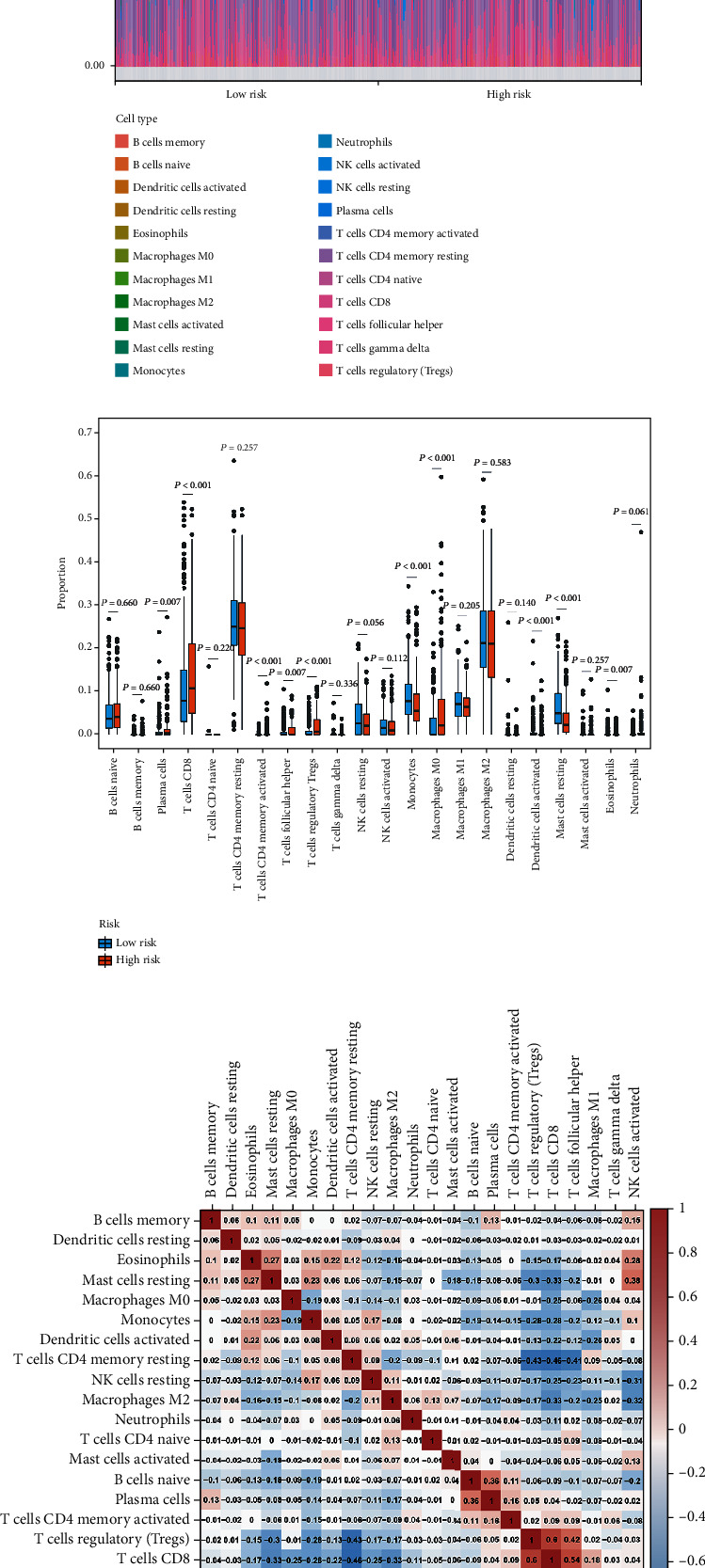
The infiltration difference of tumor-infiltrating immune cells between high-risk and low-risk groups in the TCGA cohort assessed by fourteen RRG-based prognostic signature. (a) The stacked bar chart shows the distribution of 22 immune cells in each sample of the TCGA cohort. ccRCC patients were grouped according to the median risk score. (b) Box plot shows the infiltration difference of tumor-infiltrating immune cells between the high-risk and low-risk groups in the TCGA cohort. (c) Correlation matrix of the proportion of immune cells. Red means positive correlation and blue means negative correlation.

The degree of immune cell infiltration is critical to tumor progression, treatment, and prognosis. The CIBERSORT algorithm was used to evaluate the differences in immune cell infiltration among different risk subgroups. The results showed that in each sample of the TCGA cohort, there were significant differences in the composition of 22 immune cells (Figure 9(a)). In addition, we found that there were some differences among the cells in different groups. Specifically, the infiltration degree of plasma cells, T cells CD8, T cells CD4 memory activated, T cells follicular helper, T cells regulatory (Tregs), monocytes, macrophages M0, dendritic cells activated, mast cell resting, and eosinophils were significantly different between the two groups (Figure 9(b)). Moreover, the results of correlation matrix showed that T cells CD8 had the strongest positive correlation with T cells regulatory (Tregs), and was also positively correlated with T cells follicular helper. There was also strong positive correlation between T cells follicular helper and T cells regulatory (Tregs) (Figure 9(c)).

### 3.10. Construction and Validation of a Nomogram

**Figure 10:**
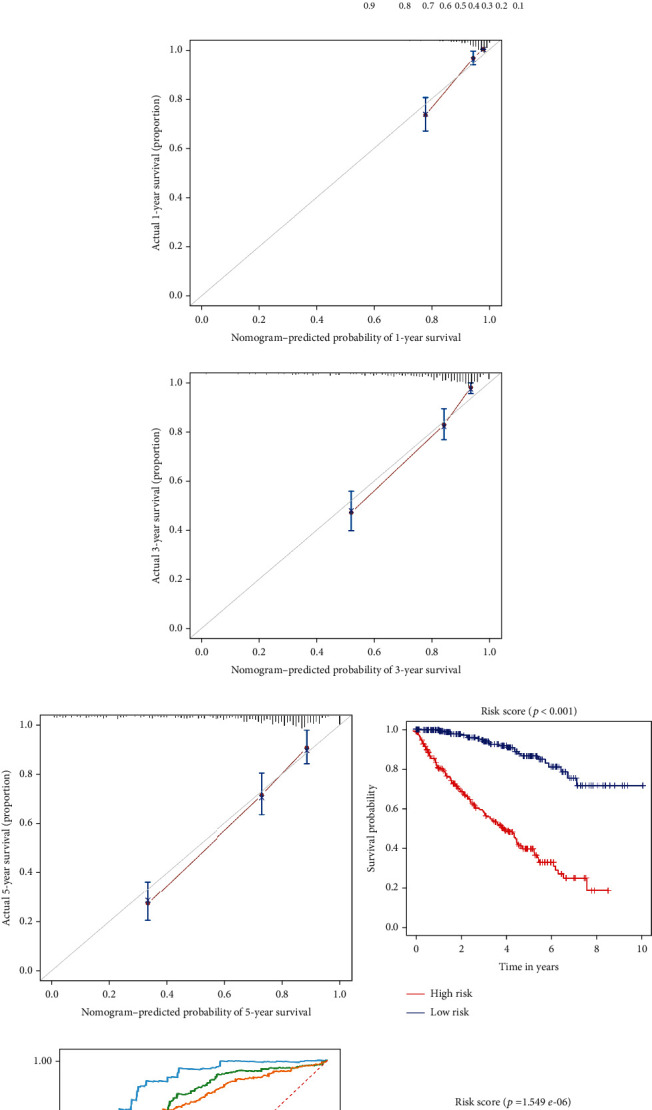
Cox regression analysis was performed on the common clinical characteristics and RRG-based signature in the TCGA cohort, and the establishment and verification of the nomogram. (a) Univariate Cox regression analysis of correlations between risk score for OS and clinical characteristics. (b) Multivariate Cox regression analysis of correlations between risk score for OS and clinical characteristics. (c) Nomogram for predicting the 1-year, 3-year, and 5-year OS of ccRCC patients in the TCGA cohort. (d, e, f) Calibration curves of the nomogram to predict OS at 1, 3, and 5 years. (g) Kaplan-Meier survival curve analysis of OS in the high- and low-risk subgroups of the TCGA cohort based on the nomogram. (h) Time-dependent ROC curves for predicting OS in the TCGA cohort based on the nomogram. The ROC curves and AUC were shown to predict ccRCC patients at 1, 3, and 5 years. (i) Kaplan-Meier survival curve analysis of OS in the high- and low-risk subgroups of the E-MTAB-1980 cohort based on the nomogram. (j) Time-dependent ROC curves for predicting OS in the E-MTAB-1980 cohort based on the nomogram. The ROC curves and AUC were shown to predict ccRCC patients at 1, 3, and 5 years.

Cox regression analysis was first performed to assess the prognostic value of different clinical parameters and risk score in ccRCC patients. The results indicated that the age (*p* < 0.001), tumor grade (*p* < 0.001), tumor stage (*p* < 0.001), primary tumor location (*p* < 0.001), lymph node infiltration (*p* = 0.049), distant metastasis (*p* < 0.001), and risk score (*p* < 0.001) of ccRCC patients were significantly correlated with OS (Figure 10(a)). However, multiple regression analysis revealed that age (*p* = 0.013), tumor stage (*p* < 0.001), and risk score (*p* < 0.001) were independent prognostic factors associated with OS (Figure 10(b)).

Subsequently, to establish a quantitative approach to predict the prognosis of ccRCC patients, we constructed a nomogram combining clinical parameters and the RRG-based prognosis-related signature by using rms package (Figure 10(c)). We mapped the points of each variable to the corresponding horizontal line and then calculated the total points of each patient and normalized it to a distribution of 0 to 100. By drawing a line perpendicular to both axes (prognosis axis and total point axis), we can estimate the 1-year, 3-year, and 5-year survival probabilities of ccRCC patients, which may be used as a reference for making clinical decisions. The calibration curve showed that the predicted value of the nomogram has a good correlation with the actual value (Figures 10(d), 10(e), and 10(f)). Moreover, to expand the clinical application and availability of the nomogram based on risk score and clinical parameters, we used TCGA and E-MTAB-1980 datasets for validation, respectively. Kaplan-Meier survival analysis showed that nomogram could better distinguish ccRCC patients with low survival rates in TCGA and E-MTAB-1980 datasets (*p* < 0.001 and *p* = 1.549*e* − 06, Figures 10(g) and 10(i)). Based on the nomogram, in the TCGA dataset, the predicted AUCs for 1-, 3-, and 5-year survival rates were 0.871, 0.804, and 0.787, respectively (Figure 10(h)), and in the E-MTAB-1980 dataset, the predicted AUCs for 1-, 3-, and 5-year survival rates were 0.897, 0.917, and 0.896, respectively (Figure 10(j)), indicating that the nomogram had good predictive power and accuracy.

### 3.11. Validation of Prognosis-Related RRG Expression

**Figure 11:**
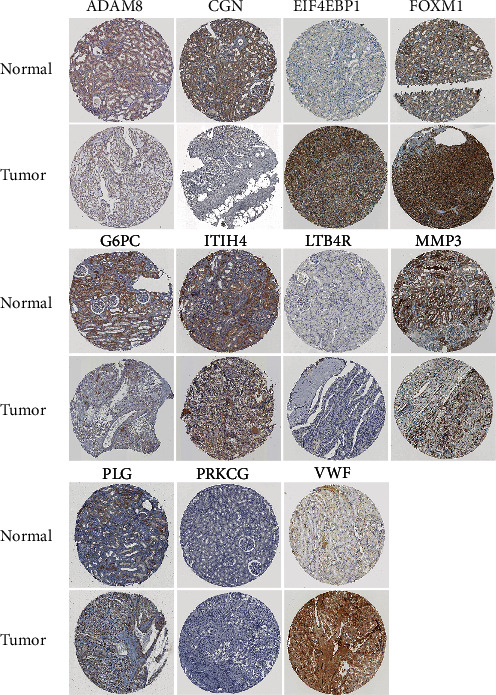
Validation of the expression of the prognostic RRGs in ccRCC and normal renal tissues in the HPA database.

We used immunohistochemical results from the HPA online database to determine the protein expression of these 14 prognostic-related RRGs. The results showed that EIF4EBP1, FOXM1, PLG, and VWF were highly expressed in renal carcinoma compared with normal renal tissue, and ADAM8, CGN, G6PC, ITIH4, and MMP3 were low in expression in renal carcinoma compared with normal renal tissue. However, there was no significant difference in the expression of LTB4R and PRKCG between normal renal tissues and renal carcinoma tissues (Figure 11) (Supplemental Table S4).

## 4. Discussion

According to the latest global cancer statistics, RCC accounts for about 3% of all cancers and is increasing at 2% per year. Approximately 99,200 new cases of RCC and 39,100 RCC-related deaths were reported in Europe in 2018 [29]. As the most common histological subtype of RCC, ccRCC is a malignant parenchymal tumor derived from renal tubular cells, with a 5-year survival rate of only 11.7% in advanced patients [30–32]. However, approximately 25-30% of ccRCC patients are diagnosed with advanced cancer, and 30% have distant metastases after surgery for early cancer [3, 4]. And the molecular mechanism is still unclear. Redox homeostasis depends on the balance between antioxidant and oxidant levels. During tumorigenesis and progression, when tumor growth exceeds the capacity of the existing vascular system to provide oxygen to tumor cells, tumor cells are often subjected to oxidative stress caused by ischemia, hypoxia, and independent anchored growth [33–35]. More and more evidence showed that redox homeostasis played a fundamental role in tumor genesis and metastasis progression [36–38]. Yet, current studies on cancer, including ccRCC, mainly focus on changes in oxidative stress. The expression pattern and role of RRGs in ccRCC is still unclear, and the redox omics characteristics of ccRCC have not been further studied.

In our current study, we identified a total of 344 differentially expressed RRGs between tumor and normal tissues based on the transcriptome data of ccRCC in the TCGA database. We systematically analyzed the biological functions and molecular mechanisms of these RRGs using bioinformatics techniques. In addition, by performing Cox regression analysis, we identified fourteen prognosis-related RRGs and constructed a RRG-based prognosis-related signature. We also explored the correlation between the prognostic signature and clinical parameters and the role of these prognostic RRGs in ccRCC. Moreover, we also explored the upstream regulatory networks of these RRGs and their relationship with immune cell infiltration.

After our thorough and in-depth analysis, we identified fourteen RRGs that were most associated with prognosis, including *ADAM8*, *CGN*, *EIF4EBP1*, *FOXM1*, *G6PC*, *HAMP*, *HTR2C*, *ITIH4*, *LTB4R*, *MMP3*, *PLG*, *PRKCG*, *SAA1*, and *VWF*. ADAM8 is a member of the disintegrin and metalloproteases family with proteolytic activity, and plays an important role in cell adhesion, migration, proteolysis, and signal transduction. High expression of ADAM8 in tumor cells has been shown to be associated with invasion and metastasis of cancer cells and is associated with poor prognosis in patients [39, 40]. CGN interactions with other proteins are involved in the regulation of tight junction assembly, cell growth, and gene expression [41]. Oliveto et al. [42] found that highly expressed CGN was a predictor of survival in mesothelioma patients, and miR-24-3p promoted tumor progression and metastasis in mesothelioma patients by inhibiting the expression of CGN. The EIF4EBP1 gene encodes a translation suppressor protein that competitively binds to eukaryotic translation initiation factor 4E, thereby inhibiting its protein expression [43]. Phosphorylated EIF4EBP1 is thought to be an indicator of tumorigenic activity and is associated with poor survival in cancer patients, while nonphosphorylated EIF4EBP1 acts as a tumor suppressor [44]. FOXM1 plays an important role in balancing genomic stability and maintaining cell proliferation and differentiation [45]. Studies have shown that FOXM1 is abnormally elevated in a variety of human malignancies and acts as a major activator of tumor cell invasion and metastasis [46]. G6PC plays an important role in the glycogen breakdown pathway. Studies have shown that glycogen plays a key role in promoting the survival of cancer cells, and inhibition of glycogen decomposition can induce apoptosis and early cell senescence [47]. HAMP plays an important role in the proliferation and metastasis of tumor cells [48]. Studies have shown that dysregulated HAMP expression is associated with an increased risk of hepatocellular carcinoma [49]. HTR2C was found to be involved in the non-small-cell lung cancer pathway, directly affecting epidermal growth factor receptor tyrosine kinase inhibitor resistance [50]. ITIH4 is an acute-phase protein secreted by the liver into the blood circulation system, and it is believed to be closely related to the occurrence, progression, invasion, and metastasis of many solid tumors. Li et al. [51] found that ITIH4 is an effective serum marker for early warning and diagnosis of hepatocellular carcinoma. LTB4R is a potent lipid mediator that regulates allergy, inflammation, and immune responses, and has been shown to be upregulated in a variety of tumors and to play a potential role in the early stages of tumor development [52, 53]. MMP3 is an extracellular matrix-degrading protease that plays an important role in a variety of tumors. Polette et al. [54] found that MMP3 expression was a prognostic marker for HNSCC invasion and lymph node metastasis. Radisky et al. [55] found that overexpression of MMP3 in breast epithelial cells was associated with epithelial-mesenchymal transformation in vitro and tumor promotion in vivo. PLG has broad substrate specificity, which not only supports the migration and invasion of tumor cells due to the enzymatic properties of fibrinolytic enzyme but also has antiangiogenesis and antitumor factors [56]. Zhao et al. [57] found that high expression of PLG in advanced high-grade serous ovarian cancer is a favorable prognostic biomarker. The PRKCG gene encodes *γ*PKC, which plays an important role in tumor genesis, proliferation, differentiation, and migration. Studies have found that mutations in the PRKCG gene increase breast cancer susceptibility [58]. Lu et al. [59] also found that PRKCG gene intron variation was significantly associated with an increased risk of osteosarcoma. SAA1 is an acute-phase high-density lipoprotein-associated apolipoprotein that is significantly upregulated in injury, inflammation, and cancer [60]. Studies have shown that SAA1 is involved in a variety of functions, including inducing extracellular matrix-degrading enzymes for tissue repair, recruiting immune cells to inflammatory sites, and lipid transport and metabolism [61]. VWF is a multifunctional adhesive glycoprotein. Elevated plasma VWF antigen concentrations have been found in a variety of malignancies [62]. Aryal et al. [63] found that intraplatelet VWF could independently predict the recurrence of early hepatocellular carcinoma after resection. These results suggested that these fourteen RRGs may be involved in the occurrence and progression of ccRCC. However, the exact molecular mechanisms are unknown, and further exploration of possible mechanisms may be valuable.

Next, we established a redox-associated prognostic signature based on these fourteen prognostic-related RRGs. Kaplan-Meier survival analysis found that patients in the high-risk group had worse OS than those in the low-risk group. ROC curve analysis showed that the prognostic signature could better screen out ccRCC patients with poor prognosis. Further analysis showed that after stratification by different clinical parameters, the prognosis of patients in each high-risk group was poor. And this prognosis-related signature was also associated with disease progression of ccRCC, and the higher the risk score, the more malignant the ccRCC tumor, suggesting that this signature has a good recognition in distinguishing the degree of malignancy of the tumor and prognosis of the patient.

In addition, we used the TCGA database to construct ccRCC network to explore the interaction between differentially expressed miRNAs and prognosis-related RRGs. A network of 9 differentially expressed miRNAs and 6 RRGs was established based on the results of coexpression analysis. These miRNAs may have the potential to activate oxidative stress or act as a great regulator of cancer triggering and deserve further investigation. To further understand the biological functions and molecular mechanisms of these differentially expressed RRGs, we performed GO and KEGG enrichment analysis. The results showed that these RRGs were significantly enriched in reactive oxygen species metabolic process, calcium ion homeostasis, antigen processing, treatment, peptide antigen presentation, HIF-1 signaling pathway, transcriptional misregulation in cancer, and PI3K-Akt signaling pathway. The imbalance of the redox system plays an important role in the pathogenesis and progression of tumors. During tumor development, when tumor growth exceeds the capacity of the existing vascular system to provide oxygen to tumor cells, tumor cells are often subjected to oxidative stress caused by ischemia, hypoxia, and independent anchored growth [33–36]. These by-products of oxidative stress cause conformational changes in DNA, proteins, and lipids that further lead to glycosylation, phosphorylation, or oxidation, thereby affecting the function and stability of biomolecules [64]. When these proteins and lipids undergo apoptosis or oxidation, antigenic changes lead to tumor resistance to radiation therapy and the host immune system [65, 66]. Additionally, excessive ROS can react with residues of various amino acids of proteins (such as cysteine, histidine, lysine, arginine, proline, or threonine) to form carbonyl groups, changing the coding sequence and tertiary and quarter-level structures of proteins [67]. These mutated peptides may produce new epitopes. These results suggest that genes may influence the occurrence and development of tumors by regulating cell redox homeostasis and affecting immune cell function. Further studies found that, based on the signature, there were differences in the degree of immune cell infiltration between high- and low-risk ccRCC groups.

Moreover, to expand the clinical application and availability of RRG-based prognostic signature and to establish a quantitative method for predicting patient prognosis, we constructed a nomogram combining clinical parameters. After drawing the calibration curve of each time cutoff point and verifying it with TCGA dataset and E-MTAB-1980 dataset for many times, it is suggested that the performance and accuracy of the nomogram are good.

Overall, our study provides new insights into the occurrence and progression of ccRCC from the perspective of redox. Our prognostic signature can better predict the survival probabilities of ccRCC patients, which may become a new prognostic biomarker for ccRCC. However, our study also has some limitations. First, our study is mainly based on a single bioomics information, and different characteristics of different platforms may lead to patient heterogeneity. Second, the model construction and validation of this study were designed by retrospective analysis, and the model still needs to be validated through a prospective clinical cohort. Finally, the specific biological function and molecular mechanism of prognostic RRGs in ccRCC are still unclear, and need to be further analyzed by *in vitro* and *in vivo* experiments.

## 5. Conclusions

In conclusion, we systematically explored the biological function and prognostic value of these differentially expressed RRGs in ccRCC by a variety of bioinformatics techniques. We also constructed redox-associated prognostic signature that could independently predict the prognosis of ccRCC patients. To our knowledge, this is the first report on the establishment of redox-associated prognostic signature of ccRCC. Our results may have important significance in revealing the mechanism of ccRCC and provide new therapeutic targets and prognostic biomarkers for ccRCC.

## Figures and Tables

**Table 1:** Multivariate Cox regression analysis to identify prognosis-related redox genes.

Gene	Coef	Exp (coef)	se (coef)	*z*	Pr (>∣*z*∣)
ADAM8	0.0632	1.0652	0.0834	0.7575	0.4488
CGN	-0.0989	0.9058	0.0569	-1.7376	0.0823
EIF4EBP1	0.1336	1.1430	0.0898	1.4880	0.1367
FOXM1	0.1039	1.1095	0.0773	1.3442	0.1789
G6PC	-0.0263	0.9741	0.0383	-0.6851	0.4933
HAMP	0.0258	1.0261	0.0595	0.4328	0.6651
HTR2C	0.1703	1.1857	0.0707	2.4100	0.0160
ITIH4	0.0460	1.0470	0.0584	0.7873	0.4311
LTB4R	0.1244	1.1324	0.0957	1.3003	0.1935
MMP3	0.0618	1.0637	0.0392	1.5764	0.1149
PLG	-0.0531	0.9483	0.0291	-1.8230	0.0683
PRKCG	0.0259	1.0263	0.0571	0.4536	0.6501
SAA1	0.0332	1.0337	0.0273	1.2146	0.2245
VWF	-0.0657	0.9364	0.0628	-1.0463	0.2954

Coef: coefficient.

**Table 2:** The relationship between prognosis-related redox genes and clinicopathologic parameters.

Gene		Age (≤65/>65)	Gender (male/female)	Grade (G1-2/G3-4)	Stage (I-II/III-IV)	T stage (T1-2/T3-4)	N stage (N0/N1-*X*)	M stage (M0/M1-*X*)
*N*		353/186	353/186	249/282	331/205	349/190	241/298	428/109

ADAM8	*t*	0.733	NA^∗^	6.708	6.059	5.807	0.995	4.203
	*p*	0.919	0.942	<0.001	<0.001	<0.001	0.640	<0.001

CGN	*t*	2.333	1.735	4.626	5.274	4.749	1.748	1.245
	*p*	0.140	0.387	<0.001	<0.001	<0.001	0.640	0.250

EIF4EBP1	*t*	2.360	NA^∗^	NA^∗^	NA^∗^	NA^∗^	0.779	NA^∗^
	*p*	0.140	0.806	<0.001	<0.001	<0.001	0.655	<0.001

FOXM1	*t*	0.638	NA^∗^	NA^∗^	NA^∗^	NA^∗^	0.869	NA^∗^
	*p*	0.919	0.530	<0.001	<0.001	<0.001	0.655	0.112

G6PC	*t*	1.027	3.144	4.643	5.276	4.730	1.144	3.983
	*p*	0.854	0.028	<0.001	<0.001	<0.001	0.640	<0.001

HAMP	*t*	NA^∗^	1.529	6.518	5.939	4.891	0.999	2.855
	*p*	0.919	0.445	<0.001	<0.001	<0.001	0.640	0.012

HTR2C	*t*	NA^∗^	0.903	NA^∗^	NA^∗^	NA^∗^	0.481	0.100
	*p*	0.229	0.587	0.019	0.355	0.324	0.680	0.920

ITIH4	*t*	0.581	NA^∗^	NA^∗^	NA^∗^	3.747	0.727	2.035
	*p*	0.919	0.636	<0.001	<0.001	<0.001	0.655	0.065

LTB4R	*t*	1.189	NA^∗^	2.676	3.679	3.906	1.086	NA^∗^
	*p*	0.823	0.587	0.009	<0.001	<0.001	0.640	0.018

MMP3	*t*	0.110	0.972	NA^∗^	NA^∗^	NA^∗^	1.351	0.854
	*p*	0.954	0.587	<0.001	<0.001	<0.001	0.640	0.424

PLG	*t*	0.258	1.314	4.076	NA^∗^	5.230	1.238	2.766
	*p*	0.945	0.529	<0.001	<0.001	<0.001	0.640	0.012

PRKCG	*t*	0.241	0.810	NA^∗^	NA^∗^	NA^∗^	0.521	NA^∗^
	*p*	0.945	0.587	<0.001	<0.001	<0.001	0.680	0.214

SAA1	*t*	0.289	2.725	NA^∗^	7.910	7.124	0.126	4.796
	*p*	0.945	0.049	<0.001	<0.001	<0.001	0.900	<0.001

VWF	*t*	0.057	0.064	3.670	3.661	3.232	0.608	3.966
	*p*	0.954	0.949	<0.001	<0.001	0.001	0.680	<0.001

NA: not available. ^∗^Nonparametric Mann-Whitney rank sum test.

## Data Availability

The data and materials can be obtained by contacting the corresponding author.

## Abbreviations

RCC: Renal cell carcinoma
ccRCC: Clear cell renal cell carcinoma
RRGs: Redox-related genes
TCGA: The Cancer Genome Atlas
ROS: Reactive oxygen species
ROC: Receiver operating characteristic
HPA: Human Protein Atlas
FC: Fold change
FDR: False discovery rate
WGCNA: Weighted correlation network analysis
LASSO: Least absolute shrinkage and selection operator
AUC: Area under the receiver operating characteristic curve
OS: Overall survival
GO: Gene Ontology
KEGG: Kyoto Encyclopedia of Genes and Genomes database
CIBERSORT: Cell-type identification by estimating relative subsets of RNA transcripts
TFs: Transcription factors.

## Supplementary Materials

Supplementary Materials Supplemental Table S1: a total of 4087 RRGs were obtained from the GeneCards, OMIM, NCBI, and GSEA-MSigDB databases. Supplemental Table S2: univariate Cox regression analysis of differentially expressed RRGs. Supplemental Table S3: transcription factors and redox genes regulatory networks. Supplemental Table S4: relevant links of immunohistochemical staining images of prognostic RRGs. Supplemental Figure S1: LASSO regression analysis for screening prognosis-related RRGs. Supplemental Figure S2: prognostic value of fifteen key RRGs in the TCGA cohort. Supplemental Figure S3: expression levels of these 14 RRGs in different cancer types in the TCGA cohort.
