# Supplementary material for: Integrated Analysis to Identify a Redox-Related Prognostic Signature for Clear Cell Renal Cell Carcinoma
Source: Oxid Med Cell Longev. 2021 Apr 21;2021:6648093. doi: 10.1155/2021/6648093 (PMC8084660; doi:10.1155/2021/6648093)
Supplement: Supplementary Materials — Supplemental Table S1: a total of 4087 RRGs were obtained from the GeneCards, OMIM, NCBI, and GSEA-MSigDB databases. Supplemental Table S2: univariate Cox regression analysis of differentially expressed RRGs. Supplemental Table S3: transcription factors and redox genes regulatory networks. Supplemental Table S4: relevant links of immunohistochemical staining images of prognostic RRGs. Supplemental Figure S1: LASSO regression analysis for screening prognosis-related RRGs. Supplemental Figure S2: prognostic value of fifteen key RRGs in the TCGA cohort. Supplemental Figure S3: expression levels of these 14 RRGs in different cancer types in the TCGA cohort. [file 6648093.f1.zip › 6648093.f1.docx]

**Integrated analysis to identify a redox-related prognostic signature for clear cell renal cell carcinoma**

**Supplemental Table S2**. Univariate Cox regression analysis of differentially expressed RRGs.

| Gene | Hazard ratio | P value | Gene | Hazard ratio | P value |
| --- | --- | --- | --- | --- | --- |
| AANAT | 1.224 | 0.001 | IL2RA | 1.181 | 0.001 |
| ABAT | 0.795 | <0.001 | IL4I1 | 1.243 | 0.002 |
| ADAM8 | 1.507 | <0.001 | ITGAX | 1.219 | 0.004 |
| AFP | 1.099 | 0.023 | ITIH4 | 1.288 | <0.001 |
| AKR1B10 | 1.089 | <0.001 | LAT | 1.233 | <0.001 |
| ALDH6A1 | 0.704 | <0.001 | LGALS1 | 1.470 | <0.001 |
| ALOX5 | 1.138 | 0.021 | LPA | 0.867 | 0.002 |
| APOA4 | 1.087 | 0.007 | LTA | 1.162 | 0.007 |
| APOC1 | 1.115 | 0.025 | LTB4R | 1.441 | <0.001 |
| AZU1 | 1.140 | 0.020 | LTB4R2 | 1.224 | 0.003 |
| BCL2A1 | 1.235 | <0.001 | LYZ | 0.910 | 0.023 |
| BDNF | 0.913 | 0.023 | MAP4K1 | 1.220 | 0.003 |
| BIRC5 | 1.493 | <0.001 | MMP3 | 1.212 | <0.001 |
| C3 | 1.114 | 0.028 | MMP9 | 1.150 | <0.001 |
| CCL11 | 1.150 | <0.001 | MT1G | 1.092 | 0.002 |
| CCL5 | 1.154 | 0.010 | MYBL2 | 1.435 | <0.001 |
| CCNA2 | 1.591 | <0.001 | NCF4 | 1.388 | <0.001 |
| CCND1 | 0.798 | <0.001 | NOD2 | 1.296 | <0.001 |
| CD36 | 0.875 | 0.005 | NOS1 | 0.899 | 0.018 |
| CDC25C | 1.396 | <0.001 | NPPA | 1.147 | 0.016 |
| CDKN2A | 1.255 | 0.002 | OCLN | 0.844 | <0.001 |
| CGN | 0.771 | <0.001 | PAH | 0.924 | 0.001 |
| COL1A1 | 1.177 | <0.001 | PCK2 | 0.824 | 0.016 |
| CSF3 | 1.127 | 0.008 | PLAU | 1.304 | <0.001 |
| CSF3R | 1.225 | 0.004 | PLG | 0.882 | <0.001 |
| CTLA4 | 1.198 | <0.001 | POU2F2 | 1.405 | <0.001 |
| CYP2J2 | 0.922 | 0.001 | PRG2 | 1.241 | <0.001 |
| DAO | 0.908 | 0.001 | PRKCG | 1.288 | <0.001 |
| DRD4 | 1.164 | 0.025 | PTGER3 | 0.884 | <0.001 |
| E2F1 | 1.270 | 0.010 | RAC2 | 1.168 | 0.025 |
| EIF4A1 | 1.234 | <0.001 | RETN | 1.125 | 0.024 |
| EIF4EBP1 | 1.511 | <0.001 | RRM2 | 1.360 | <0.001 |
| F2 | 1.115 | <0.001 | SAA1 | 1.126 | <0.001 |
| FGA | 1.041 | 0.031 | SERPINE1 | 1.128 | 0.007 |
| FGF1 | 0.852 | 0.002 | SLC11A1 | 1.428 | <0.001 |
| FGL1 | 1.096 | 0.003 | SLC18A2 | 0.824 | <0.001 |
| FOXM1 | 1.495 | <0.001 | SLC6A3 | 0.936 | 0.001 |
| G6PC | 0.859 | <0.001 | SORD | 0.856 | 0.030 |
| GATA4 | 1.109 | 0.001 | TAT | 1.133 | 0.003 |
| GATM | 0.838 | <0.001 | TDO2 | 1.131 | 0.003 |
| GCG | 1.193 | 0.040 | TF | 1.108 | <0.001 |
| GRIN2D | 1.439 | <0.001 | TFR2 | 1.136 | 0.001 |
| GZMB | 1.148 | 0.043 | TIMP1 | 1.546 | <0.001 |
| HAMP | 1.282 | <0.001 | TNFSF11 | 1.095 | 0.014 |
| HAO2 | 0.884 | <0.001 | TNNI3 | 1.118 | <0.001 |
| HK3 | 1.351 | <0.001 | TP73 | 1.165 | 0.011 |
| HP | 1.092 | <0.001 | TRPM2 | 1.299 | 0.001 |
| HSD11B2 | 0.827 | <0.001 | TYMP | 1.347 | 0.001 |
| HTR2C | 1.348 | <0.001 | VWF | 0.788 | <0.001 |
| IFNG | 1.121 | 0.003 | WT1 | 1.097 | 0.001 |
| IGFBP1 | 1.088 | <0.001 | ZAP70 | 1.216 | 0.002 |
| IL2 | 1.148 | 0.028 |  |  |  |


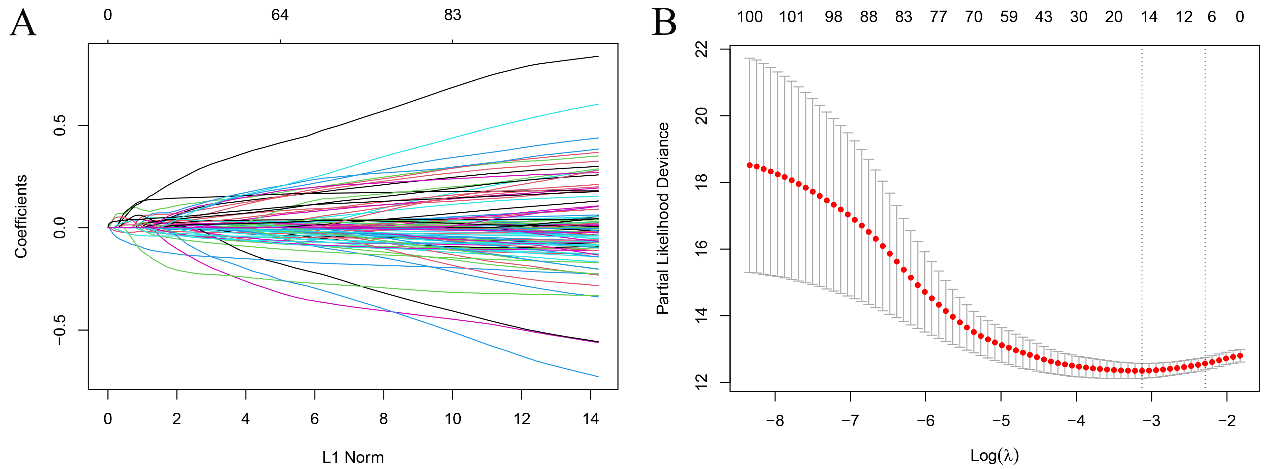


**Supplemental Figure S1**. LASSO regression analysis for screening prognosis related RRGs.

(A) Trajectories of model coefficients; (B) Cross validation fitting and performance

evaluation of the model.


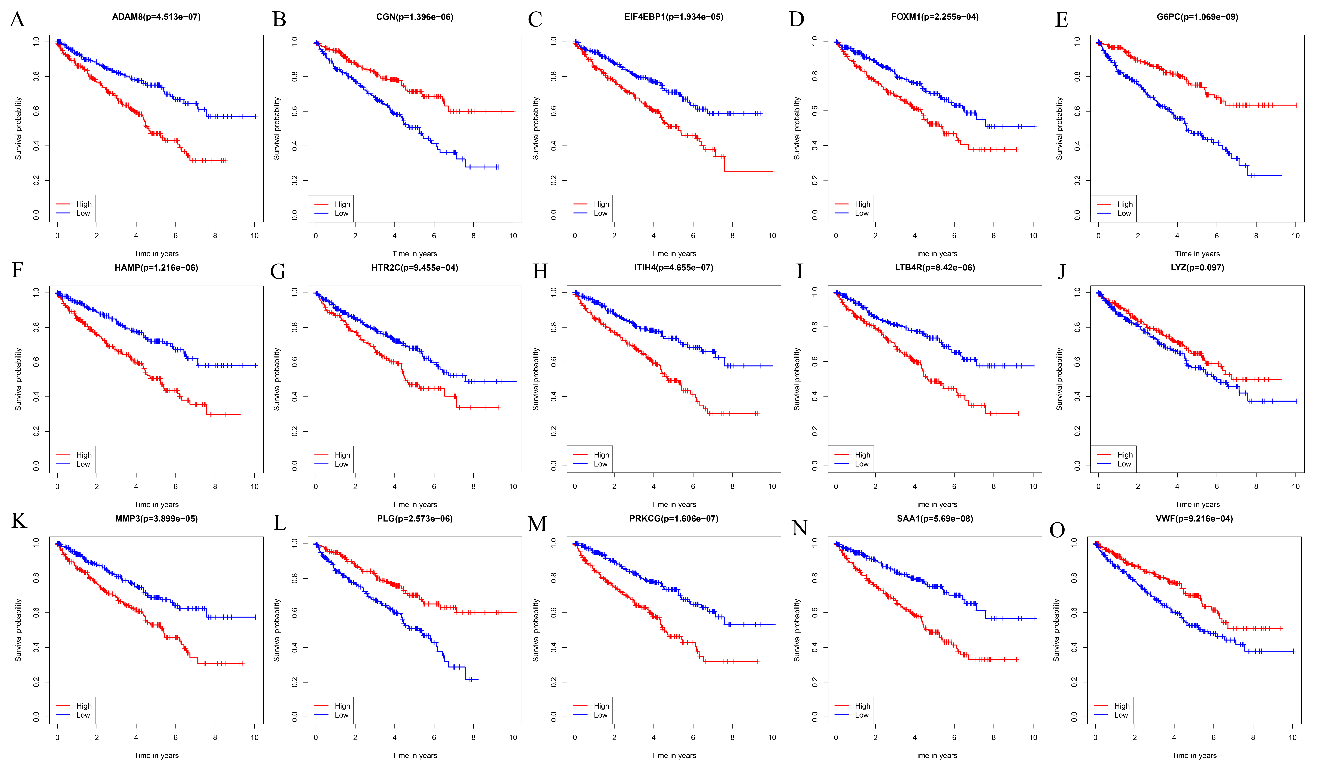


**Supplemental Figure S2**. Prognostic value of key fifteen RRGs in TCGA the cohort.

(A) ADAM8; (B) CGN; (C) EIF4EBP1; (D) FOXM1; (E) G6PC; (F) HAMP; (G) HTR2C; (H) ITIH4; (I) LTB4R; (J) LYZ; (K) MMP3; (L) PLG; (M) PRKCG; (N) SAA1; (O) VWF.

**Supplemental Figure S3.** Expression levels of these 14 RRGs in different cancer types in the TCGA cohort.
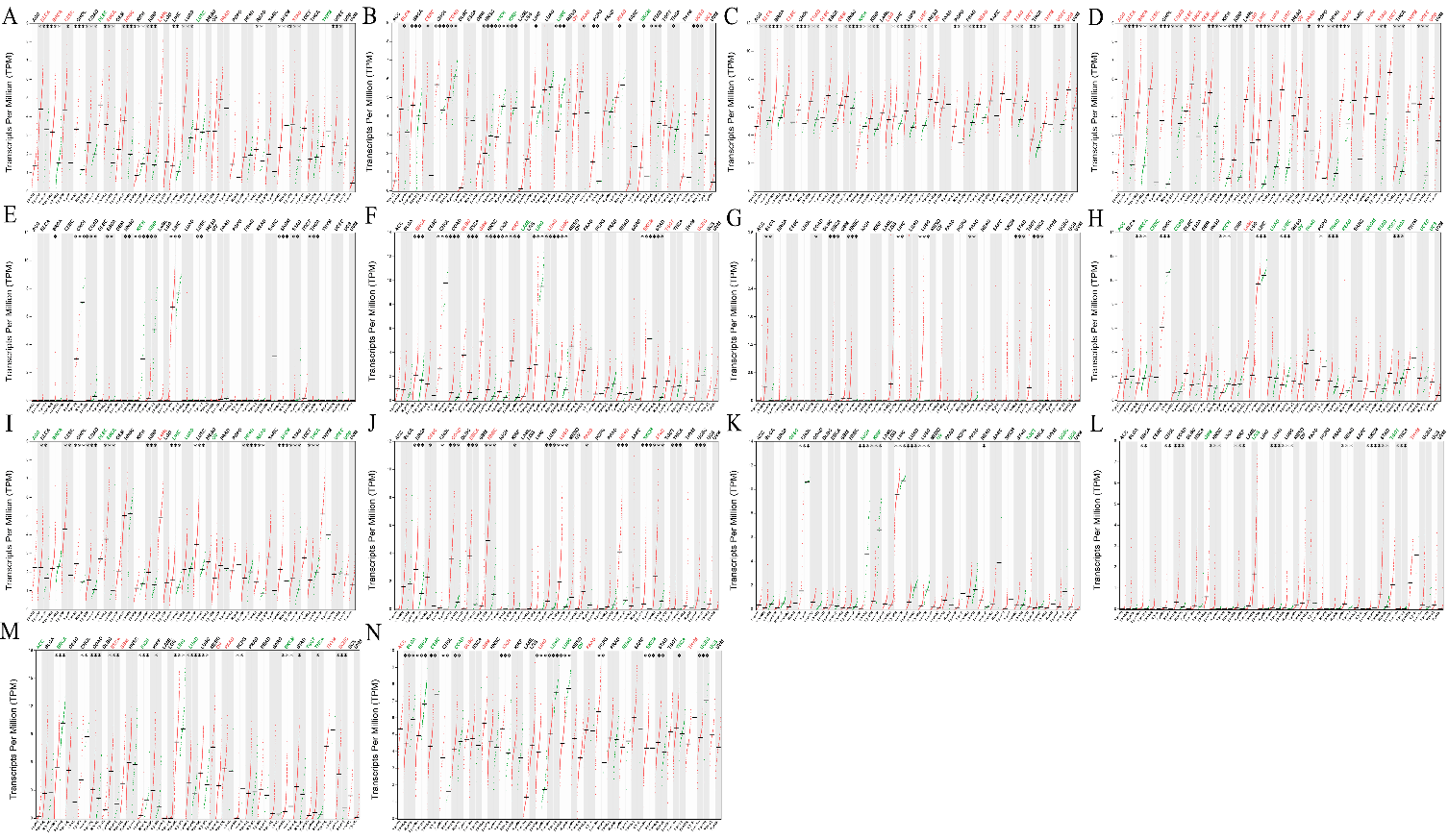


(A) ADAM8; (B) CGN; (C) EIF4EBP1; (D) FOXM1; (E) G6PC; (F) HAMP; (G) HTR2C; (H) ITIH4; (I) LTB4R; (J) MMP3; (K) PLG; (L) PRKCG; (M) SAA1; (N) VWF. ACC: Adrenocortical carcinoma; BLCA: Bladder Urothelial Carcinoma; BRCA: Breast invasive carcinoma; CESC: Cervical squamous cell carcinoma and endocervical adenocarcinoma; CHOL: Cholangio carcinoma; COAD: Colon adenocarcinoma; DLBC: Lymphoid Neoplasm Diffuse Large B-cell Lymphoma; ESCA: Esophageal carcinoma; GBM: Glioblastoma multiforme; HNSC: Head and Neck squamous cell carcinoma; KICH: Kidney Chromophobe; KIRP: Kidney renal papillary cell carcinoma; LAML: Acute Myeloid Leukemia; LGG: Brain Lower Grade Glioma; LIHC: Liver hepatocellular carcinoma; LUAD: Lung adenocarcinoma; LUSC: Lung squamous cell carcinoma; MESO: Mesothelioma; OV: Ovarian serous cystadenocarcinoma; PAAD: Pancreatic adenocarcinoma; PCPG: Pheochromocytoma and Paraganglioma; PRAD: Prostate adenocarcinoma; READ: Rectum adenocarcinoma; SARC: Sarcoma; SKCM: Skin Cutaneous Melanoma; STAD: Stomach adenocarcinoma; TGCT: Testicular Germ Cell Tumors; THCA: Thyroid carcinoma; THYM: Thymoma; UCEC: Uterine Corpus Endometrial Carcinoma; UCS: Uterine Carcinosarcoma; UVM: Uveal Melanoma. *: p-value < 0.05; **: p-value <0.01; ***: p-value <0.001.

**Supplemental Table S3**. Transcription factors and redox genes regulatory networks.

| miRNA | Redox genes | Coefficient | P value | Regulation |
| --- | --- | --- | --- | --- |
| hasmiR-3065-3p | ADAM8 | 0.216 | 6.86E-07 | Positive |
| has-miR-3065-5p | ADAM8 | 0.196 | 7.46E-06 | Positive |
| has-miR-3065-3p | HTR2C | 0.161 | 2.39E-04 | Positive |
| has-miR-3065-5p | HTR2C | 0.149 | 6.97E-04 | Positive |
| has-miR-711-3p | HTR2C | 0.149 | 6.56E-04 | Positive |
| has-miR-216a-5p | ITIH4 | 0.148 | 7.24E-04 | Positive |
| has-miR-216b-5p | ITIH4 | 0.158 | 3.03E-04 | Positive |
| has-miR-205-5p | PLG | 0.230 | 1.23E-07 | Positive |
| has-miR-30b-3p | PRKCG | 0.154 | 4.57E-04 | Positive |
| has-miR-30c-1-3p | PRKCG | 0.193 | 9.44E-06 | Positive |
| has-miR-146b-5p | VWF | 0.146 | 8.55E-04 | Positive |

**Supplemental Table S4**. Relevant links of immunohistochemical staining images of prognostic RRGs.

| Gene | Normal renal tissues | Renal carcinoma tissues |
| --- | --- | --- |
| ADAM8 | https://www.proteinatlas.org/ENSG00000151651-ADAM8/tissue/kidney#img | https://www.proteinatlas.org/ENSG00000151651-ADAM8/pathology/renal+cancer#img |
| CGN | https://www.proteinatlas.org/ENSG00000143375-CGN/tissue/kidney#img | https://www.proteinatlas.org/ENSG00000143375-CGN/pathology/renal+cancer#img |
| EIF4EBP1 | https://www.proteinatlas.org/ENSG00000187840-EIF4EBP1/tissue/kidney#img | https://www.proteinatlas.org/ENSG00000187840-EIF4EBP1/pathology/renal+cancer#img |
| FOXM1 | https://www.proteinatlas.org/ENSG00000111206-FOXM1/tissue/kidney#img | https://www.proteinatlas.org/ENSG00000111206-FOXM1/pathology/renal+cancer#img |
| G6PC | https://www.proteinatlas.org/ENSG00000131482-G6PC/tissue/kidney#img | https://www.proteinatlas.org/ENSG00000131482-G6PC/pathology/renal+cancer#img |
| ITIH4 | https://www.proteinatlas.org/ENSG00000055955-ITIH4/tissue/kidney#img | https://www.proteinatlas.org/ENSG00000055955-ITIH4/pathology/renal+cancer#img |
| LTB4R | https://www.proteinatlas.org/ENSG00000213903-LTB4R/tissue/kidney#img | https://www.proteinatlas.org/ENSG00000213903-LTB4R/pathology/renal+cancer#img |
| MMP3 | https://www.proteinatlas.org/ENSG00000149968-MMP3/tissue/kidney#img | https://www.proteinatlas.org/ENSG00000149968-MMP3/pathology/renal+cancer#img |
| PLG | https://www.proteinatlas.org/ENSG00000122194-PLG/tissue/kidney#img | https://www.proteinatlas.org/ENSG00000122194-PLG/pathology/renal+cancer#img |
| PRKCG | https://www.proteinatlas.org/ENSG00000126583-PRKCG/tissue/kidney#img | https://www.proteinatlas.org/ENSG00000126583-PRKCG/pathology/renal+cancer#img |
| VWF | https://www.proteinatlas.org/ENSG00000110799-VWF/tissue/kidney#img | https://www.proteinatlas.org/ENSG00000110799-VWF/pathology/renal+cancer#img |
