# Supplementary material for: Integrated Analysis to Identify a Redox-Related Prognostic Signature for Clear Cell Renal Cell Carcinoma
Source: Oxid Med Cell Longev. 2021 Apr 21;2021:6648093. doi: 10.1155/2021/6648093 (PMC8084660; doi:10.1155/2021/6648093)
Supplement: Supplementary Materials — Supplemental Table S1: a total of 4087 RRGs were obtained from the GeneCards, OMIM, NCBI, and GSEA-MSigDB databases. Supplemental Table S2: univariate Cox regression analysis of differentially expressed RRGs. Supplemental Table S3: transcription factors and redox genes regulatory networks. Supplemental Table S4: relevant links of immunohistochemical staining images of prognostic RRGs. Supplemental Figure S1: LASSO regression analysis for screening prognosis-related RRGs. Supplemental Figure S2: prognostic value of fifteen key RRGs in the TCGA cohort. Supplemental Figure S3: expression levels of these 14 RRGs in different cancer types in the TCGA cohort. [file 6648093.f1.zip › 6648093.f2.docx]

TableS1. The 4087 redox-related genes.

| Gene | Gene | Gene | Gene | Gene | Gene | Gene | Gene | Gene | Gene | Gene |
| --- | --- | --- | --- | --- | --- | --- | --- | --- | --- | --- |
| TXN | LDHA | BOLA2 | SLC25A13 | SMN1 | ISCU | KCNH2 | DNM2 | NAGA | USP47 | MTFP1 |
| APEX1 | UBE2I | BOLA2B | STAR | POLR2C | THBS1 | CTSK | PIK3R2 | NARS2 | ZFP36 | DNAJC4 |
| PC | CFTR | H2AC4 | KLK3 | NUDC | SPTBN1 | RORA | PLAT | S100A6 | GNLY | MRPL33 |
| GSR | DUOX1 | PDGFB | STK3 | PSMC5 | CEACAM3 | GRIA1 | KCNQ1 | PPBP | CCDC90B | MRPL52 |
| GLRX2 | KDR | RPS6KB1 | ALDH18A1 | PSMD11 | RPL14 | CPT2 | WT1 | RAPGEF4 | ACAD10 | MRPS36 |
| TXNRD2 | NAMPT | CYP11B1 | ACLY | PSMD12 | POLDIP2 | IL2RG | CASP6 | PMPCA | ACSF2 | CCDC51 |
| TXNRD1 | NQO2 | SLC25A4 | CKB | PICK1 | PIK3CA | RPS19 | CACNA1B | TFAP2C | FASTKD2 | CYSTM1 |
| NMRAL1 | CYP21A2 | RRM1 | HADHA | PTPRM | SPTAN1 | TUBA1A | FGG | TFE3 | CST7 | SERP1 |
| GLRX | EPO | SLC11A2 | DFFA | UBE2K | MYH10 | GHSR | ANPEP | VARS2 | HINT2 | IFNA14 |
| P4HB | LOC107372315 | RAB11A | EHMT2 | UBE2S | FLNB | GJB2 | ADRB2 | AKR7A2 | COX7C | IFNA16 |
| GPX1 | CXCR4 | TKT | PDHB | RASSF2 | ACOX1 | GLDC | ACE2 | ABCB8 | HEY2 | PRELID2 |
| PRDX5 | ITGB3 | CRYAB | NR1I2 | KIDINS220 | NFS1 | MC1R | CYP2C8 | GCAT | CLSTN1 | SMDT1 |
| NFE2L2 | SCO1 | CFH | NPHS1 | RAB1A | SLC2A4 | BMP2 | CDKN1B | MIPEP | CIRBP | SLN |
| CISD1 | CA9 | AOC3 | IDH3A | PTX3 | GPD1 | MITF | GRIN2D | GLUD2 | HMGN2 | PLGRKT |
| PRDX6 | AGTR1 | ARF6 | PPM1A | RAB5C | ELK1 | DLG4 | CALCR | MARS2 | MTPAP | SLC25A34 |
| HMGB1 | CA2 | GADD45A | HSD17B10 | TLN1 | RPS3 | DDX3X | PRKCI | ATP13A2 | MRPL15 | SLC25A48 |
| SELENOT | ITGA4 | MUTYH | ISL1 | PSMD3 | GPC1 | CSNK2A2 | PDPK1 | LTA | MRPS2 | TMEM143 |
| TXNRD3 | BCAT1 | IL13 | TOP2B | PSMB6 | RPS2 | IFIH1 | NR2F2 | FBXW11 | MRPS28 | TRIM4 |
| PRDX1 | INTS6 | BMP6 | STUB1 | DCTN2 | RPS20 | PIK3C2A | HTR2C | FANCF | MUL1 | LGALS7B |
| TXNL1 | RAC2 | BCAR1 | TDO2 | CIB1 | RPS26 | PROS1 | NOD2 | AK3 | DUSP23 | TTC23 |
| PRXL2A | TXNDC15 | LIF | DLST | EBF1 | RPS13 | MSX2 | OPRM1 | BMP3 | MYL7 | TAMM41 |
| NXN | NHLRC2 | DDAH1 | ADM | RANGAP1 | RPS17 | YAP1 | SLC12A6 | FKBP8 | PDP2 | TMEM242 |
| PRDX2 | VASN | APOA4 | ATP6AP2 | RAB1B | RPL31 | F9 | SPTLC2 | ACSL5 | PDPR | TMEM134 |
| SELENON | GRXCR1 | HBA1 | AHSG | GOLGA2 | RPL13 | ALPP | TNNI3 | FOXE1 | NRBF2 | PSTK |
| SOD1 | DNAJC16 | CDIPT | ABCD3 | CBX1 | RPS9 | FST | KCNJ2 | MAP2 | SH3PXD2B | COX8C |
| TXN2 | LDHB | IMMT | BRD4 | BAG2 | NFU1 | CLTC | KCNN4 | ATXN2 | SLC7A3 | TIMM21 |
| HIF1A | CCL11 | DAP | CNDP1 | AP3D1 | RPLP0 | MSR1 | IL6R | CTSE | SLC25A26 | ZCCHC24 |
| SELENOO | MTRR | FBXL5 | DYNC1H1 | AP3S1 | RPL7 | SLC25A20 | RUNX1 | HOMER2 | SLC25A27 | ZNF580 |
| TXNIP | MSRA | GPX6 | NDUFV2 | FARSA | RPS4X | SIGMAR1 | POMC | CPT1C | SLC25A28 | ZBED5 |
| PARK7 | SMG1 | ZBED1 | IL12B | AMOT | RPL23 | PTGER3 | VRK1 | CLCN5 | POFUT2 | ATP5PF |
| PRDX4 | COQ9 | MT-CO2 | PFKFB3 | AK4 | RPS25 | UNG | CARM1 | HNRNPD | SLC30A6 | CYB561D1 |
| CAT | IDH2 | IGF1R | SDHD | ERLIN2 | TCF19 | TCF4 | CAPN2 | CHIA | SLC25A42 | RIDA |
| NOX5 | TH | MAP2K2 | RPL35A | BPTF | MIR21 | GPD2 | GK | CERK | SLC25A25 | MRPL58 |
| PRDX3 | FASLG | AR | OGT | MDC1 | MAP2K1 | WNT1 | CCR3 | HBG1 | SHPK | MT-ND4 |
| NOX4 | ANXA5 | FLT1 | SLC17A5 | ACTR1A | JAK2 | C1QBP | MAP4K4 | MTO1 | NPRL3 | MT-ND5 |
| SELENOH | HLA-B | PRKG1 | ITPKB | CPVL | CTSD | CCR1 | LRP1 | DPEP1 | IQCE | DUSP28 |
| SELENOW | LOX | HSD11B1 | PYCR2 | AP1M1 | FYN | AQP4 | ANTXR2 | DUT | ISCA1 | NAXD |
| JUN | HPX | MYH9 | IL3 | HDLBP | PAH | FDPS | ATP1B1 | MOCS2 | TOMM34 | CCDC58 |
| KEAP1 | IDH1 | DUSP6 | NDUFA10 | DNAJA2 | GNAI2 | ANGPTL4 | ERCC2 | ELAC2 | LGALS4 | MRM2 |
| PDILT | COMT | REN | PLAUR | EEF1E1 | ALDH1A1 | BCL2L11 | ADORA2B | NDUFAF4 | TET1 | SDHAF3 |
| CBS | KCNMA1 | ATF6 | USP1 | EEF1G | ACP5 | BMP1 | ADSL | NDUFB3 | SURF4 | TMEM223 |
| SOD2 | CD36 | ATP7B | GDI1 | EFHC1 | TRAF6 | BCKDK | ACTA1 | IL16 | TMEM70 | PRR5L |
| NQO1 | HSP90AA1 | EPHX2 | GNE | MOB1A | IRF1 | MDH1 | FOLR1 | PDSS1 | PHYKPL | NME1-NME2 |
| ERO1B | F3 | DIABLO | ANP32A | EIF3H | PRKCB | ABCD1 | CTSH | PRRX1 | DRG2 | KYAT3 |
| GLRX3 | EIF2S1 | SPHK1 | AGTR2 | NOP10 | PDP1 | ACACB | CDKN1C | SFRP2 | MYO5C | ARMS2 |
| RNF7 | CCL5 | OAT | ACAA1 | PQBP1 | ITGAL | HPD | HADH | SERPINB3 | NADK2 | ATP5PB |
| ERO1A | NRF1 | TFAP2A | GSTO1 | SMARCA5 | TUFM | GSTM3 | DIAPH1 | IDUA | HAO2 | ATP5MG |
| CHCHD4 | FOSB | GLS | MMACHC | PITPNB | DNM3 | HNRNPA2B1 | CD28 | PROCR | HIGD1A | MTARC2 |
| HMGB3 | PGAM1 | F2R | NDUFS6 | POLR2H | HSD17B1 | HNRNPK | DDX58 | SDC3 | MRS2 | DEFB4B |
| TMX1 | AHCY | CTSL | EIF5A | NUP93 | MT2A | EEF1A2 | COL3A1 | NME4 | TIMM44 | MRM3 |
| CYCS | IGF1 | CNTN2 | HTRA1 | NXF1 | CSF3 | NEDD4 | CYSLTR2 | PNPT1 | CAND1 | ELSPBP1 |
| GPX4 | SDHB | SIK2 | PAX4 | PHF6 | BOLA3 | PPP2R2B | CSF3R | LDHC | GATC | NIPSNAP2 |
| TMX3 | HBB | PXN | SLC37A4 | SRP72 | BOLA1 | IDH3B | CD79B | TREX1 | GHITM | NRDC |
| SELENOV | COX4I1 | RAB5A | SLC25A11 | PSMC6 | MT-CO1 | IGFBP7 | RHO | TST | CCL26 | AKR1B15 |
| ARNTL | DCXR | HSP90B1 | PSME2 | PSMD1 | DBH | OPRK1 | PDHX | RARS2 | AURKAIP1 | FAM210A |
| PDIA2 | AKR1C1 | TARDBP | TSFM | PSMC2 | EIF4E | IL6ST | PPP2R1B | KCNMB1 | ATPAF1 | ATP5MC1 |
| HMGB2 | ME1 | AASS | TAGLN | TPD52 | SERPINE1 | RAN | HTRA2 | KLK6 | ATPAF2 | ATP5MC2 |
| NOS3 | GSTM2 | MTHFD1 | IYD | UFM1 | MEF2C | KPNA2 | PRKACB | KPNA1 | ARPP19 | ATP5ME |
| SCO2 | DNAJA1 | OPA1 | TFAM | KCNS3 | YES1 | KLF4 | PRKACG | RAB11B | EXOG | MRPL57 |
| MPO | AHSP | NADK | LRPPRC | TAX1BP1 | MAP2K6 | KRT19 | PAX3 | SUMO3 | ARMC4 | CCDC167 |
| UQCRFS1 | IL6 | NAT1 | AARS2 | RCC1 | CYP27A1 | TWIST1 | SOX2 | TK2 | FAHD1 | TOMM70 |
| HMOX1 | MDM2 | CYP51A1 | COX15 | RBM4 | LEP | XPO1 | SCN1A | PDCD10 | FAM136A | TEX19 |
| CLOCK | TLR4 | DUSP4 | EEF1D | RBM8A | ACADSB | ALOX5AP | SCN2A | LGMN | LYRM7 | TMEM256 |
| NPAS2 | HNF4A | KCND2 | RPN1 | STX5 | ELANE | FABP4 | SCN3A | EIF2AK1 | ADO | ANP32C |
| NMRAL2P | CYP1A1 | HYOU1 | SNCG | DHX30 | TRPV1 | BAK1 | SCN8A | EARS2 | ADPRH | FDX2 |
| TNF | EPHA3 | XRCC1 | TRIM21 | PPA1 | GPC4 | FADS1 | SCN9A | NAGS | AIFM3 | H19 |
| FDX1 | RELB | TNFRSF8 | CUL2 | RBBP7 | CTNS | LTB4R | SCNN1A | DHTKD1 | METAP1D | MTERF2 |
| TP53 | TOR1A | CALB1 | TFEB | ZC3H14 | SDC4 | ALDH4A1 | SLC6A4 | PMPCB | ACOT13 | MT-ND4L |
| EPAS1 | EEF1A1 | BBC3 | CCK | CACYBP | TUBB4B | MCM7 | SMAD2 | TFB1M | ACOT2 | MTARC1 |
| G6PD | RNASE1 | S100A1 | ADNP | MAGED2 | TRPC4 | FUS | HSPA9 | THPO | ADHFE1 | DELE1 |
| XDH | CYP2D6 | BLZF1 | BMP15 | ADRM1 | CUL1 | CPT1B | HSD17B3 | XRCC2 | ACSM2A | OXLD1 |
| GFER | UCHL1 | DEFB1 | FLOT2 | AHSA1 | GNB4 | HNF1B | UBE2D3 | YME1L1 | ADCK1 | SLX4IP |
| MAPK8 | CASP7 | TOR1AIP1 | BHMT | AASDHPPT | FANCG | DGUOK | UBE3A | ADCYAP1 | BTBD10 | INTS11 |
| NOS1 | VIM | GABPB1 | BRD3 | ACTL6A | HTT | RPL15 | ITPA | ALOXE3 | C1GALT1C1 | AFG1L |
| MICAL1 | TNFRSF1A | ORAI2 | CHKA | CXCL14 | SDC1 | NDUFS2 | CYP24A1 | FLCN | RMND1 | CEMIP2 |
| CASP3 | SLC16A1 | INTS10 | MRPS22 | CYP2W1 | PON2 | NONO | CYP27B1 | CARS2 | CLGN | COA8 |
| PTPN1 | AKR1C3 | TOR1AIP2 | MRE11 | HPS5 | GLS2 | SDHC | POLB | CAPN10 | HEPHL1 | CFAP74 |
| ACP1 | PDK1 | H2BC21 | EIF5 | CPSF6 | DDB1 | NLRP1 | PLA2G6 | GJA3 | GLOD4 | DGLUCY |
| CYBB | CUL3 | GGT2 | COPA | COPZ1 | SETX | RPS10 | OTC | GLYAT | GUF1 | DMAC2L |
| ALDH5A1 | PARP2 | NOTCH3 | PPIC | HNRNPDL | SDCBP | NTF4 | PPARD | LTK | COX14 | SERPINA2 |
| TXNDC17 | ANGPT1 | MAP3K7 | S100A11 | HNRNPH3 | RPS27 | TPM2 | KRT5 | BBOX1 | COPS6 | PRELID3A |
| MAPK14 | HNF1A | PIK3R1 | NFKBIB | CGN | RPLP2 | KMT2A | WNT3A | APLP2 | COMTD1 | SDHAF4 |
| ATF4 | HMGCR | MYD88 | OSGEP | CHMP2A | RPS15A | KITLG | LIPE | LTB4R2 | CHAC1 | SSC4D |
| PDIA3 | CEBPB | TGFB2 | SND1 | MTA2 | SRF | PTRH2 | GAMT | ATAD1 | CHD6 | MICOS10 |
| PIR | SECISBP2 | SYK | PTPRT | NAP1L1 | MAFG | SF3B1 | GLP1R | ASAH2 | DNAJB4 | TCAIM |
| POR | TRDN | ATP1A1 | UBE2M | NAP1L4 | AKAP12 | PBRM1 | AQP2 | ALOX15B | DNAJC15 | MAIP1 |
| GLO1 | ROMO1 | FLNA | PTP4A2 | DDX23 | MYOF | THBD | ETV1 | ANO1 | DNAJC27 | BICDL1 |
| TXNDC11 | FTH1 | RIPK1 | TRMT1 | DDX39B | PPL | LIAS | ALDH6A1 | AGK | MTHFD2L | FMC1 |
| TMX2 | CYP11A1 | EDNRB | XRCC3 | EIF3C | MT1A | VNN1 | BIRC3 | AGPAT5 | MRPL17 | ETFRF1 |
| NOS2 | FDXR | CPT1A | GNL3 | EIF3D | UTRN | WARS2 | ABCB6 | MCAT | MRPL19 | HDHD5 |
| DNAJC10 | COX17 | GSN | CCT7 | EIF3I | PELO | VTN | C5 | BCAP31 | MRPL24 | MTRES1 |
| CLIC4 | ABCG2 | IL2RB | EPS15L1 | RPL32 | SNTB2 | ARPC3 | CEL | ACOX3 | MRPL28 | DEPP1 |
| CISD2 | NPM1 | PPP1CA | FKBP2 | COPE | AQP8 | ANXA6 | DMD | FOLR2 | MRPL40 | DMAC2 |
| MPST | PRKCA | PGK1 | MBD2 | IFNA1 | MPRIP | ARHGEF7 | GPT2 | FOXC1 | MRPL42 | PRELID3B |
| SENP3 | AKR1C2 | AKR1C4 | CYBRD1 | SNX2 | RPL38 | BCKDHB | GATA1 | CA3 | MRPL46 | PEDS1 |
| GPX7 | TNFSF10 | C3 | GSTK1 | SAP18 | COA6 | ADH1B | NDUFS7 | FCAR | MRPS23 | RAB5IF |
| SLC8B1 | CFLAR | GALT | DNAJB11 | SAV1 | RPL22L1 | FMO1 | NEDD4L | CYB5R1 | MRPS25 | MIR126 |
| TXNDC2 | ACHE | CTNNA1 | ECI2 | RPL36 | KBTBD4 | CDX2 | NEFL | CYB5R2 | MRPS34 | MIR27A |
| TXNDC8 | DUSP3 | MTHFR | IL19 | NFKBIE | DCAF11 | COQ7 | NR4A2 | CLDN5 | DHRS1 | FAM215A |
| CLIC1 | KCNA5 | MUC1 | PCMT1 | INA | ITPRID2 | DLG1 | NME2 | GK2 | MRPS6 | H2BS1 |
| MAPK1 | XRCC6 | CYP2A6 | SART1 | PSMD13 | METTL26 | MYO1E | HSD3B2 | GSTA1 | DPM2 | TMEM35B |
| DLD | SLC23A2 | PFN1 | HSPB2 | PTMA | PCNA | RPL18 | SLC25A12 | GSTA2 | MTCH1 | MIR141 |
| CLIC2 | PTGIS | PLD2 | UBE2O | TPSB2 | DHFR | RPL21 | SLC12A5 | COX4I2 | DUSP15 | MIR34B |
| NGB | MFN2 | SLC4A1 | SUMO2 | SYMPK | STK11 | NAT2 | SLC22A5 | COX8A | ECHDC1 | MIR34C |
| SELENOF | APAF1 | LBR | PECAM1 | STOX1 | FGF1 | DBT | TPT1 | HIBADH | DEFA6 | ANXA2P2 |
| GCLC | CD81 | RUNX2 | PARP3 | RAB9A | FANCA | NDUFA9 | TYMP | CLDN10 | DEFB4A | MIR122 |
| MIEN1 | DUSP1 | BECN1 | JMJD1C | STK26 | CYP3A4 | SFPQ | LIG1 | HACE1 | MRM1 | RIPOR3 |
| NDOR1 | TRPA1 | CFI | PSMC4 | NCBP1 | NMNAT1 | SIRPA | KYNU | HCCS | NDFIP1 | MIRLET7A1 |
| LGALS9 | HSPA6 | PTGS1 | CD53 | OTUB1 | EIF4EBP1 | SELENBP1 | KAT5 | MTFMT | NDUFA7 | MIR17 |
| UQCRC2 | CTTN | FCER2 | CKMT1B | POLR3K | PINK1 | IFNGR2 | SUCLA2 | MRPL3 | IFNA5 | MIR200B |
| RNH1 | PPIF | ETFB | CHCHD2 | PIP4K2C | ACADS | IGFBP1 | PYGM | MORF4L1 | HTRA3 | MIR206 |
| HRAS | SAMHD1 | DDOST | MRPL13 | IRS4 | TYRP1 | IGFBP4 | IL5RA | DECR1 | NMRK1 | MIR93 |
| UQCRC1 | HSPA1A | EIF4G1 | MYBBP1A | WTAP | DAO | SNW1 | ALAS2 | DARS2 | NIT1 | MIR342 |
| MACROH2A1 | ETHE1 | S100B | DAP3 | GEMIN4 | PON1 | NUP214 | CAMKK2 | RPL3 | PLA2G15 | AFG3L1P |
| COX19 | NOX3 | NFATC2 | NES | LUC7L | FOSL1 | SLC39A14 | GNMT | NDUFA8 | SLC25A33 | MIR378A |
| GPX3 | TOMM40 | PTPN12 | SFXN3 | FIP1L1 | KNG1 | SLC39A4 | GJA8 | NDUFAF1 | OMP | GGT3P |
| SLC7A11 | ERP29 | IKBKE | DHRS9 | AGTRAP | ENDOG | PTPN7 | F8 | NDUFB10 | NUDT6 | MIR590 |
| SLC52A1 | ABL1 | LAT | MEMO1 | ACOT8 | UCP3 | ITM2B | FANCL | NEDD8 | SLC25A16 | MIR455 |
| TPI1 | SLC2A1 | HSPG2 | ABCE1 | HNRNPAB | SP3 | TIRAP | LRP2 | RPL34 | SLC25A23 | PCGEM1 |
| CYBA | MDH2 | ITCH | DNAJC7 | RNPS1 | SLC25A10 | VAPA | FHL1 | PFKFB4 | NUDT15 | MIR1-2 |
| DHODH | KDM1A | CCNA2 | MRPS17 | EIF3B | FLAD1 | XRN2 | MAF | PDHA2 | SLC15A3 | MIR30D |
| RAC1 | LMNA | CBR3 | MRPS9 | EIF3E | CYP46A1 | CCS | ATIC | SFXN4 | ILVBL | MIR520D |
| VKORC1 | DPYSL2 | BSG | NACA | EIF3G | CD63 | EXOSC3 | MAD1L1 | SEL1L | PTGR2 | MIR675 |
| CDKN3 | STAT5A | PABPN1 | KARS1 | IK | DCT | ANKRD1 | AMACR | SH3KBP1 | PHLDA2 | LINC01619 |
| HAO1 | ELN | RPL35 | GOLGB1 | PAG1 | AGR2 | ACTC1 | AMT | IFNA2 | PHPT1 | MIR15A |
| MAP3K5 | SERPINB2 | S100A4 | ARL6IP5 | RSL1D1 | OXT | ACTR3 | ALDH1A3 | PARS2 | TPPP3 | SNORA12 |
| COQ2 | MTTP | CBX3 | ABHD10 | NOLC1 | IAPP | COQ6 | ALDH3A2 | IARS2 | UGGT1 | DUX4L1 |
| ERP44 | NUMA1 | AGPS | BTG3 | SLMAP | RPS8 | DMGDH | ADRB3 | PARL | UGGT2 | VTRNA1-1 |
| RYR1 | TRAP1 | CRY1 | CKMT1A | ILF2 | YBX1 | NCL | AGXT | IFITM3 | TSHZ3 | VTRNA1-2 |
| CXCL8 | BANF1 | TJP1 | MRPS35 | RCVRN | DDX39A | EIF4G2 | ERG | PPM1K | STARD7 | ISCA1P1 |
| CYC1 | ID3 | EGR2 | SP140 | PSMD6 | BAIAP2L1 | HNRNPU | MECP2 | RYR3 | RAB32 | FAM136BP |
| FOXO1 | DEK | VDAC2 | PLP2 | TP53INP1 | ENC1 | PPP1R12A | ABAT | SDHAF2 | KIAA1549 | AOX3P |
| NFE2L1 | CLEC4A | BNIP3 | TAF5 | MYCBP | MIR155 | PREP | ABCC3 | SARS2 | TCF20 | TXNP1 |
| NCF1 | IKBKB | HBA2 | DUSP12 | DSC1 | STAT1 | SAA1 | ACAD8 | RTN4IP1 | RDH14 | GLRXP2 |
| NOX1 | MYB | NDUFA4 | SUB1 | EIF3K | ADAM10 | RPS7 | ACAT2 | ENSA | SUPV3L1 | TXNP5 |
| NR1D2 | ANXA1 | SEPHS1 | ZC4H2 | SCAMP3 | HGF | SARDH | MGLL | NMNAT2 | SUN2 | TXNP6 |
| MB | SMAD3 | MFN1 | ATAD3B | TIGAR | TLR2 | NFIX | C1S | SLC7A8 | SH3BGRL | GLRXP1 |
| GAPDH | PLD1 | CYB5B | AHNAK | USO1 | SLC1A3 | RPL7A | ATP2C1 | SLC25A32 | SAMM50 | TXNP2 |
| ATP5IF1 | ABCC2 | COX5B | MCU | VAT1 | PGR | PNKD | CTLA4 | SLC30A5 | PACRG | TXNP4 |
| LPO | PIN1 | MPC1 | BRF2 | FLG2 | ITGB2 | SLC31A1 | ERCC6 | OPLAH | PCDH1 | CHCHD2P8 |
| APP | CYP2C19 | NDUFA5 | GRPEL2 | GATAD2A | BRCA2 | NUP98 | CLCN2 | SLC25A21 | REXO2 | LOC727947 |
| GOT2 | ETFA | PABPC1 | MRPL14 | CCT8 | HDAC3 | NTSR1 | MMAB | NT5C | TFB2M | HBB-LCR |
| TXNDC5 | DHRS4 | PLS3 | MRPL2 | MARCKSL1 | PRKCQ | LASP1 | CHRNA1 | SEMA3E | TIMM8B | MSBP1 |
| PTS | TPH1 | LETM1 | MRPL23 | BASP1 | QDPR | TRPC1 | ROS1 | SLC22A8 | TIMM9 | TXNP3 |
| CYB5R4 | TPR | VSNL1 | DERL1 | API5 | GCDH | TCOF1 | E2F4 | SIL1 | ZDHHC6 | HDL3 |
| PITRM1 | EPX | CCT2 | MRPL49 | ACOT9 | GOT1 | PYY | DDB2 | SSH1 | ZNF3 | LOC171417 |
| UQCR10 | HAGH | G3BP1 | MRPS11 | CBLC | FANCD2 | KRT10 | NDUFS8 | PSME1 | VPREB1 | LOC117134604 |
| FOS | RPA3 | SMPD2 | MRPS21 | CAPRIN1 | CYP11B2 | GCG | NDUFV1 | SRM | ZADH2 | LOC117134605 |
| ITPR1 | PTPRU | TAGLN2 | MRPS31 | CDK11A | MSN | MAP4 | PDK4 | TOP1MT | CARD16 | LOC117134606 |
| GPX2 | SUOX | HSPA1B | PFDN2 | COPS7B | TALDO1 | F2RL2 | SERPINI1 | TRIT1 | GATB | LOC117134607 |
| KRIT1 | PRKAB1 | HSPE1 | HSD17B13 | COPS8 | KRT8 | FBXO7 | OXCT1 | TRNT1 | GARS1 | LOC117134608 |
| LIPT2 | BCR | CCN2 | TOMM22 | CKAP4 | GGCX | FANCE | PISD | TRPC5 | MICU2 | LOC117134611 |
| H6PD | ABCB1 | ATAD3A | TRMT10C | CHMP4A | GPI | BCL3 | IGFBP3 | TRPM3 | ANO9 | LOC117135104 |
| UQCRB | MMP3 | SFXN1 | HSPA13 | MLEC | SLC40A1 | FLII | PCBD1 | REV1 | METTL7B | LOC117135105 |
| ATG4A | PLAU | NFYA | YARS1 | DNAJB5 | ITGAM | FOSL2 | PCCA | TCP1 | BLOC1S1 | LOC117135106 |
| UQCRQ | RARA | TNPO1 | YEATS2 | DERL2 | SPP1 | ERCC8 | PRKG2 | KLC1 | ABCA9 | LOC110599569 |
| ATG4B | PRMT1 | RASD1 | ZBTB33 | DHX8 | AVP | HNRNPC | PRF1 | TERF1 | ABHD11 | AASDH |
| UQCRH | SET | KCND1 | MARS1 | NAA50 | MAP2K7 | MLXIPL | RPS6KA5 | SULT4A1 | ADCK5 | AASDHPPT |
| UQCR11 | PIK3CB | FUBP1 | MGME1 | CD2BP2 | APOB | DUSP2 | NGFR | SUCLG2 | ACAD11 | ABHD10 |
| AQP11 | VHL | GPX5 | CXorf56 | MOB1B | ERN1 | DUSP5 | PLA2G2A | KCNE2 | AVEN | ACACA |
| DNAJC24 | BAD | SSBP1 | SARS1 | DAZAP1 | CXCL12 | MYL6 | PML | TKTL1 | RMND5A | ACACB |
| CYB561D2 | REL | MRPL12 | TOX4 | NDFIP2 | NDRG1 | MPV17 | SLC19A1 | KSR1 | APOC4 | ADH4 |
| PDIA6 | CD38 | MPC2 | RARS1 | ELP2 | SAT1 | RNF13 | SEMA3A | PRIM1 | GRSF1 | AGT |
| EGF | PTPRA | SEPHS2 | TBPL2 | HNRNPUL1 | SIRT5 | GRP | INPP5D | TP53I3 | RILP | AIFM1 |
| VEGFA | TP73 | CHCHD3 | PPARGC1B | SPIN1 | GZMB | CSRP1 | PTPRB | POLI | COQ10B | AKR1A1 |
| RYR2 | CR2 | CSN2 | SCML2 | PRPF19 | GP6 | CRY2 | PHYH | KCNK2 | DPY19L3 | ALKBH1 |
| CCL2 | OCLN | FYB1 | HSDL2 | SERPINB10 | CSF2 | PAPPA | TIMP3 | ST8SIA2 | MTRF1 | ALS1 |
| SETD2 | CPOX | MRPS14 | PGAM5 | NTPCR | CST3 | PASK | LARS2 | POLRMT | MTFR1 | APEX1 |
| BCL2 | PTPRS | SQOR | MRPL50 | SMPD4 | HAMP | PCBP1 | TUBA4A | KIF3A | MRPL16 | ARMD1 |
| HTATIP2 | TRIM28 | SELENOK | ZNF280C | PLRG1 | GRIN2C | SNRPE | ULK1 | VAMP8 | MRPL18 | ARNTL |
| RNF41 | STIP1 | LOC110973015 | ERVW-1 | NUDT21 | IL18 | RPS15 | ITGB5 | VRK2 | MRPL20 | ATG4A |
| LACC1 | CNBP | LOC111365141 | EMC2 | SRP19 | PLXNB1 | RPS3A | STT3A | ULK2 | MRPL32 | ATORS |
| CBSL | CTRL | ERBB2 | ATP5PD | SRP68 | KCNN3 | RPS5 | TCF3 | WWTR1 | MRPL39 | ATPIF1 |
| CYB5A | PTPN11 | PPP3CA | CISD3 | PTMS | ASIC1 | PTGES | TCIRG1 | LIPT1 | MRPL43 | BLVRA |
| GGT1 | MYC | HDAC6 | ATP5MF | SRRT | TRAF4 | RAB35 | IVD | MAT2B | MRPS10 | BMI1 |
| FAS | FTL | PTPRC | GATD3A | IST1 | SURF1 | KHSRP | SUCLG1 | GDF11 | MRPS18A | BRAF |
| DDIT3 | CYGB | ACVRL1 | IGHE | STRAP | GJC1 | TRPV2 | PRKD2 | GNG5 | MRPS18C | CARD19 |
| QSOX1 | SELE | ACTN1 | GAS5 | SUPT16H | CD163 | RPL23A | TRPM4 | B3GALT4 | MRPS24 | CBS |
| ICAM1 | CREBBP | CAPN1 | FGFR1 | TMOD2 | ADH1C | PPA2 | MYH6 | FBXO32 | MRPS27 | CCS |
| NNT | SCN5A | LPL | BLK | DYNC1LI1 | UBE2E3 | TFF3 | RAC3 | BACH2 | MRPS30 | CGDX |
| PDIA4 | MAPK12 | LRRK2 | RAD50 | DCTPP1 | GP1BB | KRT7 | HTR1B | FGL1 | MRPS33 | CHCHD4 |
| ALB | TAP1 | HDAC1 | YWHAG | GZMK | AMBP | ABLIM1 | PCCB | APOC1 | DHRS7 | CISD1 |
| NDUFA2 | CDK1 | RB1 | F10 | EIF3M | MTHFD1L | APLP1 | ITPR2 | FGF20 | DUSP18 | CISD2 |
| MT-CYB | FBP2 | XIAP | ATP2A1 | POLDIP3 | PPIG | AGXT2 | ITPR3 | LSM4 | DUSP26 | CISD3 |
| GSTP1 | IL18R1 | B2M | BLM | RAB21 | LCP1 | FKBP3 | TIMP1 | AS3MT | DUSP8 | CLOCK |
| HSCB | SNCB | PRKCZ | GALK1 | THOC1 | POLG2 | CRYGD | VAPB | ASGR1 | MYL10 | CMTX4 |
| AKR1B1 | TRADD | NTRK1 | HPRT1 | THRAP3 | NFIC | CTDSPL | LMX1B | ASGR2 | EEFSEC | CNR1 |
| IL1B | SLC3A2 | TPM1 | PDHA1 | ZC3HAV1 | HNRNPL | CAPZA1 | ADIPOR1 | ALDH1L2 | EFHD1 | COQ2 |
| NCF2 | RBX1 | TBP | EGLN1 | GAR1 | TRIM22 | COTL1 | AFG3L2 | BPHL | MOBP | COQ6 |
| NAPRT | PSMD10 | STIM1 | ENO3 | CCAR2 | SCG5 | CKAP5 | GFM1 | MAVS | DCAKD | COX20 |
| PTEN | ACBD3 | VWF | PIP5K1C | CCDC80 | IFI6 | CHDH | FA2H | ABCB10 | EI24 | COX7C |
| SOD3 | NOP58 | MCM2 | TOP1 | EPRS1 | IPO11 | DIO3 | ANXA4 | ABCD2 | NDUFB5 | COXPD29 |
| MIF | MRTFA | MCM4 | STXBP1 | EPDR1 | KYAT1 | ECH1 | FARS2 | ACYP2 | IFNA6 | CPT1A |
| NFKB1 | EP300 | ALDH7A1 | VCP | ERH | ATP5F1E | CD276 | ASPA | ADAMTS17 | PAK5 | CTBP1 |
| OMA1 | ANXA2 | FPR1 | YWHAB | AARS1 | PSEN1 | IGF2BP1 | FBLN5 | AADAT | SNPH | CTBP2 |
| HBG2 | HSPD1 | CYP2B6 | MAT1A | CUTA | RAD51 | NME6 | ALDH1B1 | ACSM1 | PRELID1 | CYB561A3 |
| STAT3 | TNFRSF1B | CYP3A5 | MAT2A | CRNKL1 | CDKN2A | RPLP1 | MCCC1 | ACP6 | PRICKLE4 | CYBB |
| SMPD3 | ADAM17 | POU5F1 | APRT | CPSF7 | DNMT3A | RPS16 | MCCC2 | FN3K | SCARA3 | CYGB |
| TGFB1 | SPR | SCP2 | FGA | CLCC1 | NOTCH1 | RPS18 | ME2 | FOXRED1 | SCCPDH | CYP17A1 |
| CP | MAP3K1 | TPM3 | BUB3 | COLGALT1 | GSK3B | NTS | MECR | C1GALT1 | RPUSD4 | DDIT3 |
| NADSYN1 | CYP2E1 | TLR8 | MALT1 | CHORDC1 | MME | TIMP4 | FKBP4 | CRYBA1 | RSAD1 | DEE82 |
| MAPK10 | CALM1 | RAP1A | LYZ | MTPN | DNMT3B | STEAP1 | BCKDHA | CROT | NFXL1 | DEFB1 |
| SNCA | JUND | TGFB3 | HEXB | DNAJC9 | ITGB1 | STK25 | ACSL1 | GTPBP3 | NIPSNAP3A | DJ1 |
| PARP1 | DHDH | VCL | MTAP | DARS1 | JAK1 | SNRPD2 | FOXC2 | COX7A2L | OSGEPL1 | DNMT3A |
| SHC1 | INTS4 | LMNB1 | GRN | DCD | COL1A1 | KRT2 | GAL | COX7B | SLC25A35 | DNMT3B |
| NUP62 | GSTT1 | CCR5 | CSNK2B | COPG1 | PRKDC | LMO7 | G6PC | COQ3 | SLC25A40 | DUSP1 |
| MTR | CAV1 | ACTN4 | PPP2R1A | SERPINB12 | PHGDH | BDH2 | MAD2L2 | HIC1 | NUTF2 | EI24 |
| PRNP | GCH1 | NDUFS3 | IMPDH2 | SEC16A | TRPV4 | MIOX | CX3CR1 | DNAJA3 | SLC39A2 | ENC1 |
| FKBP1B | ACADM | NRG1 | UBE2N | SAP30L | ASS1 | FIS1 | CXCL10 | DGCR8 | NT5M | ENOX1 |
| EGFR | POU2F1 | HSD17B4 | YWHAH | SARNP | ACTA2 | AKAP1 | CYP2J2 | DIO1 | PHYHIPL | ERAF |
| HSPA4 | NR2E3 | TLR7 | ARF1 | PHF5A | ACACA | AKAP10 | HPSE | DIO2 | TONSL | ERO1L |
| SELENOS | ATP5F1A | MCM3 | ASNS | PSPC1 | IKBKG | ACMSD | CTBP2 | DMRT1 | TMTC1 | ERO1LB |
| CDKN1A | RET | EZR | FEN1 | LARS1 | IDE | GAN | APOA2 | MT1X | TMEM14C | ERVW1 |
| CDC25C | FLT3 | BID | FOLH1 | TNRC6C | SLC1A2 | GUCA2B | GPR37 | DUSP16 | TOMM7 | FAM213A |
| SIRT2 | TOP2A | ABCC8 | CES1 | UPP2 | TRPC6 | COX6C | HK3 | GAST | LACTB | FAM49B |
| AKT1 | YWHAE | CDK9 | DRD4 | TSPAN4 | YY1 | HNRNPM | COX6A1 | MPP1 | LACTB2 | FECH |
| BAX | RRM2B | MYH14 | DKC1 | TTC4 | GDNF | HNRNPR | CLPP | DEGS1 | LANCL1 | FLAD1 |
| RELA | CACNA1C | NDUFS1 | PRKAR2A | SURF6 | CCNB1 | DHRS2 | HMBS | NDUFS5 | LETMD1 | FOXO3A |
| GSS | MAPK9 | EMD | PAX5 | TMF1 | GJB1 | RPL18A | CKM | NDUFV3 | LECT2 | GAPDH |
| VCAM1 | DES | HNRNPA1 | UBA1 | TSPAN32 | F7 | CDC5L | CHMP2B | NDUFAB1 | PYROXD1 | GCLM |
| MSRB1 | YWHAZ | SERPINH1 | SUMO1 | NCOA5 | BACE1 | EHD2 | MYO5B | NDUFAF2 | SUGCT | GDPD5 |
| NFKBIA | GFAP | ST14 | PSMA6 | SCYL2 | EPHX1 | RPL24 | CD9 | NDUFAF3 | TMEM230 | GLRX2 |
| AIFM1 | FADD | UBE2L3 | PRPS1 | TIMM13 | HK2 | CRELD1 | CASQ2 | NDUFB4 | DUS2 | GOT1 |
| SRC | BDNF | TMPO | GNB1 | WBP11 | CDH5 | SIPA1L3 | CXCR3 | NDUFB6 | MRPL10 | GOT2 |
| ARHGDIA | KCNB1 | POLG | F2RL1 | YTHDC1 | NPC1 | SF3A1 | NDUFB9 | HPR | DHX29 | GPD1 |
| HVCN1 | HLA-A | ADD1 | ABCB11 | EXOSC6 | SLC18A2 | RPL6 | EHHADH | CD69 | KCNK17 | GPD2 |
| PRKAA1 | MOG | MCM5 | CPS1 | MACROD1 | TUBB1 | RPS11 | EIF4A1 | GRIN3B | JAGN1 | GPX4 |
| ATOX1 | PSMD4 | CCR6 | HINT1 | DDX19A | IRF3 | SLC25A37 | NDUFA6 | PEX11B | RAB24 | GRPEL1 |
| MMP9 | PSMD14 | ANGPT2 | DSG2 | SEC22B | IRS1 | SRSF7 | NEU1 | IL27RA | THEM5 | GRPEL2 |
| ARNT | ELAVL1 | ADAR | MYH11 | PDCL3 | KCNJ5 | SRSF9 | SKP2 | PDLIM1 | TIMM22 | GSK3B |
| CASP9 | PSMC3 | RNASEH1 | SAE1 | SAFB2 | VAV1 | RBP1 | SERPINF1 | PDSS2 | TIMMDC1 | GSR |
| CHUK | PSME3 | RICTOR | SLC5A5 | RRP12 | ALAD | MT1F | SERPINF2 | SFXN5 | ZNF346 | H6PD |
| ATM | BCLAF1 | CRYAA | SMARCB1 | PHRF1 | CASP4 | ZNF638 | IDI1 | OXR1 | CABP2 | HAAO |
| MTOR | GOLGA3 | SHMT2 | SMARCE1 | UBAP2L | FKBP1A | VIL1 | SOCS3 | IFRD1 | CCL15 | HSD11B1 |
| CYP17A1 | KRAS | PPP3CB | SMC3 | LDHAL6A | BTRC | YBX3 | PAX7 | S100P | CCDC136 | HYOU1 |
| TF | ITGA2B | RPTOR | PMVK | TTBK1 | CNR1 | UBD | SCN4A | NHLRC1 | FAM162A | IDH2 |
| ALOX5 | GLUL | PGRMC1 | UBE2D1 | KCTD12 | DDX5 | ZNF143 | NLRP12 | NFE2 | FITM2 | IMDDHH |
| EGLN2 | FASN | STMN1 | SRPK1 | RALYL | HMGCL | CBR4 | RPS14 | NLRX1 | ARMC1 | ITPR1 |
| AGT | ACE | PYGB | PCSK1 | RBM23 | NPPA | LYRM4 | SLC7A5 | SLC25A29 | ARMC10 | KEAP1 |
| HMOX2 | TRPC3 | LONP1 | EIF2AK2 | TMED9 | SLC25A1 | LRRFIP2 | PNPLA2 | PITX3 | LRRC8D | KYNU |
| ESR1 | MAP2K4 | BMI1 | MYO6 | TMED4 | ITGAV | BMS1 | HSD17B2 | OS9 | ADCK2 | LCN2 |
| NDUFS4 | CRP | MCM6 | TGIF1 | SF3B6 | AFP | MRPS12 | SLC25A15 | HSD17B8 | CPQ | LYN |
| MMP1 | UCP2 | GSTA4 | UROD | HTN3 | GATM | MYL12B | NTHL1 | NUDT2 | HEMK1 | MAP3K5 |
| EDN1 | CS | PAM | USP14 | IARS1 | FH | DEFA5 | SLC12A7 | SLC25A14 | CNN3 | MFM8 |
| PTPN3 | S100A8 | NUP107 | WRN | COPS4 | ARG2 | DDX24 | NPPB | SLC25A18 | COX18 | MICAL1 |
| DUSP19 | AIFM2 | IQGAP1 | CASP14 | H3-3A | ATF2 | RNLS | TOLLIP | NUP153 | CMC1 | MIEN1 |
| TAPBP | INTS5 | PICALM | CALM2 | HRNR | ADCY10 | NMNAT3 | UGT2B7 | NT5C3A | HMBOX1 | MIR1-1 |
| HSF1 | GJA1 | KPNB1 | APOH | PRPF38A | CSTB | SLC31A2 | LGALS3 | PHB2 | CPEB2 | MTCO1 |
| INTS2 | MAPT | KIF5B | ATP6V1A | NOC3L | EIF2S3 | SRP14 | LIG3 | PIGH | CHERP | MTCO2 |
| SP1 | CTH | ID1 | ATP6V1B1 | QPCTL | SHMT1 | ILF3 | ITGAX | PTPRR | MMUT | MTCO3 |
| INTS3 | PRKAA2 | USF1 | FMR1 | ZNF32 | S100A10 | SSBP3 | STS | L2HGDH | MTRF1L | MTCYB |
| AKR1A1 | FGF2 | UBC | CTPS1 | FOXN2 | HSD11B2 | SYNCRIP | SYN1 | SYNJ2 | DNAJC11 | MTF1 |
| TYR | PGD | VDAC3 | GANAB | CWC15 | TLR9 | TDRKH | KHK | TBPL1 | MTG1 | NADSYN1 |
| CD44 | BLVRA | GDF2 | AP1B1 | CWC22 | XBP1 | SF3B2 | REV3L | TDP2 | MTG2 | NAPRT |
| PTP4A1 | CYP1A2 | LTC4S | HMGA2 | MMGT1 | XPC | EML4 | KMO | RECK | MRPL21 | NDUFA12 |
| BCL2L1 | SLC25A3 | COPS5 | CHD4 | CHTOP | ALAS1 | EXOSC4 | TCN2 | STC2 | MRPL22 | NDUFS4 |
| HSPA8 | IL2 | EDEM1 | MTM1 | H3-3B | ARSB | CEBPZ | RBP4 | RAB6A | MRPL27 | NEDMCMS |
| PAX8 | SELP | EEF1B2 | DGKG | MOB2 | ATP2A3 | HNRNPA3 | KL | RAB8A | MRPL30 | NFE2L2 |
| CREB1 | PPARA | CDH13 | RPA2 | DPY19L1 | ATF3 | COX7A1 | KIF1B | RAB8B | MRPL35 | NFYC |
| PTGS2 | MBP | MLST8 | EIF2B2 | HNRNPUL2 | FGF4 | DSPP | SULT2A1 | RAD9A | MRPL36 | NMNAT1 |
| HSPA5 | HAAO | NFYC | NCOA1 | SFSWAP | ALDH3A1 | GRK2 | RALBP1 | IL32 | MRPL37 | NMNAT2 |
| GLUD1 | TUBA1B | GZMA | NCOA2 | INIP | AGER | DDX27 | SREBF1 | INF2 | MRPL4 | NMNAT3 |
| BCAT2 | MTF1 | AAAS | PSMA4 | RABL6 | FOXM1 | GRIN3A | DUSP10 | IL33 | MRPL48 | NNMT |
| ACO1 | MAFK | MTCH2 | PEBP1 | SEPTIN2 | MYO5A | SIPA1L1 | MTHFS | LIMS1 | MRPS15 | NNT |
| GPX8 | DNAH8 | GPT | PPM1B | ATAD3C | DDIT4 | NUP35 | EIF2AK4 | SDSL | MTIF3 | NOS1 |
| SMPD1 | COQ10A | CAPZA2 | SOAT1 | LSM12 | TRAF2 | NUP205 | NDUFA12 | S100A7 | MRPS26 | NOS3 |
| PTK2 | SELENOM | PTBP1 | RUVBL1 | FAM98B | UBE2D2 | LGALS7 | NDUFA13 | SNAP91 | MXRA8 | NOX1 |
| SDHA | METAP2 | CAPZB | RUVBL2 | AKAP17A | KCNQ4 | STAU1 | HMGCS2 | THG1L | GLYATL1 | NPAS2 |
| EGR1 | SLC1A5 | CCL4 | NHP2 | CENPV | WNT2 | MT1H | CFL2 | TIMM17A | DEDD | NQO1 |
| FMO2 | INTS9 | GORASP1 | NSF | CLDN17 | AKR1B10 | RPL17 | CUL5 | VRK3 | DDX28 | NQO2 |
| APOE | LYN | RPL30 | TAP2 | H3-4 | BDH1 | DOCK5 | RNASEL | UXS1 | MPV17L2 | NUDT1 |
| RHOA | TSC2 | PRG2 | TMED10 | H3C2 | GHRL | SF3A3 | RAPGEF3 | LMOD1 | NDUFAF6 | NXN |
| VKORC1L1 | ACTB | NFYB | HUWE1 | HNRNPA1L2 | EWSR1 | RACK1 | TFR2 | GFM2 | IFNA21 | P4HB |
| ORAI1 | PSEN2 | NLN | PRPH | RBMXL2 | ATG7 | IMP3 | USP10 | MGAT5 | IBA57 | PBD1A |
| SIRT1 | CYP2C9 | SSRP1 | MAP4K1 | TMEM109 | MGST1 | PGP | YARS2 | AZU1 | OXNAD1 | PDHA1 |
| LGALS1 | NR3C2 | TPM4 | AP1S2 | ZNF512 | C5AR1 | RCL1 | VAMP1 | GGT7 | IFNA13 | PDILT |
| KIR2DS4 | TPO | RCN2 | ETF1 | CCDC124 | LOXL1 | G3BP2 | LITAF | MICU1 | PDE12 | PDK1 |
| PDGFRB | NME1 | PPP1R15A | ATP6V0D1 | H1-0 | COX10 | FTSJ3 | ADIPOR2 | ARNTL2 | NIPSNAP3B | PDK2 |
| MAOA | TNFSF11 | AGFG1 | FBL | H3C3 | CPA6 | COQ8A | ACSF3 | MANF | SLC48A1 | PDK3 |
| XRCC5 | TNFRSF11B | ACIN1 | AIMP1 | H3C4 | PPOX | DHRS11 | ALPI | ANKRD26 | SLC25A44 | PDK4 |
| BACH1 | PPARGC1A | HOXB5 | ACTR2 | KPRP | LCN2 | DUOXA1 | CAV3 | AIF1 | SLC25A47 | PDSS1 |
| QSOX2 | MTHFD2 | CORO1C | FOXO4 | CAAP1 | ASPH | NOP2 | GPAM | AGMAT | OCIAD1 | PGAM5 |
| ATP2A2 | NUDT1 | RHOD | FSCN1 | MACROH2A2 | ATP8A1 | NPM3 | GMPPB | BCL2L13 | SPRYD4 | PKM |
| BIRC5 | RPL10A | EDF1 | CYP4A11 | H2BC4 | ADAM8 | PRODH2 | BAG1 | ME3 | PIGY | PNKD |
| NUP155 | SELENOP | DSTN | CLCN4 | H2BC5 | COMMD1 | LAMTOR5 | AUH | ABCC12 | PTCD3 | POR |
| AHR | PRKCD | CCL3 | HAX1 | H3C10 | CETN2 | TMBIM6 | MAP1LC3A | ABCG4 | TRIAP1 | PRDX1 |
| CTSB | ACTG1 | RPL28 | DSG1 | H3C12 | IL17A | RBM14 | ARRB1 | AANAT | UQCC2 | PRDX2 |
| IFNG | PRKCE | PDLIM5 | CD82 | KNOP1 | SLC30A8 | ARPC5L | APOC3 | ACSM3 | TBRG4 | PRDX4 |
| CD4 | APOA1 | SESN1 | DDX1 | PBDC1 | SRI | MCUR1 | ARHGAP1 | ACSM5 | TATDN3 | PRDX5 |
| PIK3CG | CYP1B1 | PPP1R9B | EIF2B1 | H1-10 | RAD23A | RRP15 | LRPAP1 | ACSS1 | JCHAIN | PRKAA1 |
| IL4 | CSK | RRBP1 | EIF4A3 | HDGFL2 | SELL | SLC25A30 | EPM2A | FASTK | TMEM176A | PRKG1 |
| COX5A | TSC1 | TMOD3 | RPL26 | H2AZ2 | CLCN3 | OSGIN1 | MBTPS1 | ATP10D | TMEM11 | PTGES |
| ALDH9A1 | TGM2 | S100A12 | CORO1A | H2BC10 | DNASE1 | VARS1 | MBTPS2 | CRLS1 | TP53I11 | PTPN1 |
| AQP9 | IL10 | ARPC4 | PRKCSH | H2BC6 | RPL19 | ARSH | MCEE | CTF1 | DUSP11 | PTPN11 |
| FMO5 | PLCG1 | MICAL2 | IL21 | H3C11 | PER1 | ACTBL2 | MCFD2 | CD5L | CHCHD7 | PYROXD1 |
| CTBP1 | PRODH | COX7A2 | NOP56 | H3C6 | NPC2 | PPTC7 | BCS1L | GRPEL1 | NPAT | RAC1 |
| MT3 | ITGA2 | HEXIM1 | SEC23A | H3C7 | IFNB1 | RRS1 | MDK | COX11 | DACT2 | RNF7 |
| ADH5 | HSP90AB1 | DDX21 | POU3F2 | H3C8 | ICMT | TMEM33 | ACAA2 | COX20 | TIMM10B | RNH1 |
| SUMF1 | SLC25A5 | IL27 | PPM1G | H2AC7 | RPL4 | PDCD11 | ACAD9 | RHOT2 | ZNF622 | ROMO1 |
| ERP27 | ALOX12 | SAFB | PDE3B | PPIAL4A | TTPA | NOL7 | ACADL | COQ4 | YIF1A | RSMD1 |
| CANX | DUOX2 | LARP1 | POLR2E | RTRAF | UBIAD1 | ATP5F1B | GALC | COQ5 | LYRM1 | RYR1 |
| OLR1 | E2F1 | STOML2 | PLP1 | H2AC8 | RERE | AHNAK2 | CA5A | CLYBL | CTU1 | SCARA3 |
| PTK2B | MYO1C | TWF1 | SLC25A6 | H2BC11 | PXDN | FOXRED2 | FPGS | HERPUD1 | GTPBP8 | SCO1 |
| GSTM1 | PEX5 | EDC4 | OLA1 | H2BC7 | PRIM2 | H1-4 | CXADR | CLPX | RHBDD3 | SCO2 |
| PKM | PLEC | ALYREF | PTGES3 | H2BC8 | UTS2 | PRRC2C | CXCL1 | CHCHD10 | COA3 | SELENON |
| GABPA | ITIH4 | HP1BP3 | PSMD7 | H3C15 | MAFF | SLTM | CYP4B1 | CERKL | MTERF1 | SELENOP |
| NR3C1 | TG | CSTF3 | UBA2 | POLR1G | ATF7 | SRRM2 | APOD | MRPL44 | MRPL34 | SELENOT |
| ETFDH | MSRB2 | MYO18A | PTPN13 | H3C13 | ALDH1L1 | LARP4 | EXO1 | MTIF2 | MRPL45 | SELENOW |
| SGCB | JUNB | RPL26L1 | RBBP4 | QARS1 | FLVCR1 | RBM34 | CD47 | MRPS7 | MRPL51 | SENP1 |
| PDIA5 | TGFB1I1 | IFI27 | RANBP1 | SPINDOC | CXCL2 | COQ8B | RHOQ | MRRF | MRPL9 | SESN1 |
| TFRC | MYL12A | SAP30BP | RCAN1 | PALM2AKAP2 | DHX9 | MT-ND6 | RHOT1 | MTX1 | ECHDC3 | SESN2 |
| VDAC1 | DBN1 | RALY | KIF1A | MIR200C | CD93 | VOPP1 | COX6B1 | MTX2 | NAXE | SFRP2 |
| IREB2 | GSX1 | TBCA | TECR | H2AC19 | DDX17 | MCMBP | HNF4G | ECSIT | NDUFAF7 | SH3BGRL2 |
| CALR | ALDH2 | IL4I1 | RAB10 | PEDS1-UBE2V1 | MOCS1 | RMDN1 | CMPK1 | NDUFC2 | GRINA | SH3BGRL3 |
| GCLM | PTPN6 | FUBP3 | PSMD2 | MIR661 | PDLIM7 | CGB3 | HIBCH | NDUFB2 | IFNA4 | SHC1 |
| INSR | ETS1 | MRPS18B | POLR1C | MIR1246 | PRDM2 | H4C1 | CLPB | NEIL1 | IFNA8 | SHPK |
| SORD | ESR2 | P4HTM | HSPH1 | LOC111589215 | PLEK | SELENOI | HFE | GPR65 | IFNA10 | SLC25A30 |
| NME8 | PLA2G4A | INTS8 | LMNB2 | DDC | SLC30A1 | PATJ | CKMT2 | HOGA1 | NOA1 | SLC2A10 |
| LCK | RHEB | LRRC59 | CALM3 | CASP1 | LDHD | TMEM241 | MLYCD | PRX | SLC25A2 | SLC2A4 |
| ABCC1 | NR1H2 | MICAL3 | ANXA7 | MGMT | S100A2 | MROH7 | CGA | PELP1 | NUDT19 | SLE |
| CYB5R3 | EIF2AK3 | SEPTIN9 | MBD4 | DGKE | ABCF2 | ATP5MC3 | HARS2 | PRSS2 | SPTSSA | SOD1 |
| SIRT3 | CD40LG | SERBP1 | ATXN10 | SOS1 | HEPH | H1-3 | MRPS16 | SFXN2 | PTCD2 | SRXN1 |
| RPA1 | PPIA | RTCB | CNP | IL4R | RFK | MT-CO3 | MSMO1 | PCBD2 | INTS12 | SUMF1 |
| TRPM2 | JUP | PNN | MTA1 | PTPN22 | SLPI | SEPTIN4 | MSRB3 | NIF3L1 | TRIM56 | TIMELESS |
| BLVRB | CLU | MT1G | DTYMK | TGFA | SLC39A1 | CYRIB | MYBPC1 | NIPSNAP1 | JPH4 | TMTC1 |
| OXA1L | HDAC9 | CSN1S1 | CALD1 | ZBTB16 | TOMM20 | CARD19 | EBP | OPA3 | TMEM120A | TMTC2 |
| DNM1L | ADIPOQ | SNU13 | EIF3F | ANG | UBE2E2 | TERC | RPIA | SLC25A31 | NARS1 | TMX2 |
| NCF4 | ALOX15 | ATP5PO | NEFM | NR4A3 | KPNA6 | H4C14 | CD86 | SLC9A9 | STING1 | TMX3 |
| TXNDC12 | PDGFA | SMCP | PSMA2 | HTR3A | FTMT | SEPTIN11 | GRK4 | HSPB7 | YBEY | TMX4 |
| NXNL1 | UCP1 | RBM27 | PRPF4 | PDE2A | HNRNPH1 | H2BC13 | GRK5 | PMAIP1 | ADM2 | TP53 |
| TMX4 | TSPO | CGB5 | PAICS | P2RX7 | CETN1 | MIR200A | SPG7 | OGDHL | GET3 | TP53I3 |
| SQSTM1 | FGF7 | H3C1 | PALLD | THBS2 | CIAPIN1 | NCF1C | PDK2 | SLC25A36 | CCDC12 | TRPC1 |
| RANBP2 | PTPA | FABP12 | PDCD6IP | ADH4 | NOXA1 | MIR433 | PER2 | SLC25A39 | B3GLCT | TRPC5 |
| CSNK2A1 | ATP5F1C | SEPTIN7 | SMN2 | HIPK2 | KLHL41 | MIR205 | SI | SLC25A46 | ASIC5 | TRPM2 |
| ODC1 | CTNNB1 | H3C14 | SENP1 | USP2 | PAM16 | RN7SK | SFRP4 | SLC52A3 | ACSM4 | TXN |
| RPS27A | NFKB2 | H2BC1 | NSUN2 | MYBL2 | CYP20A1 | MIR382 | SFTPB | NUBPL | C15orf48 | TXN2 |
| ORAI3 | MMP14 | GET1 | PSMC1 | GRK1 | TIMM10 | LOC110467515 | HTR2B | PGRMC2 | CYB561A3 | TXNDC12 |
| SLC2A10 | IRAK1 | H2AC18 | SSB | GPD1L | GADD45GIP1 | LOC107548112 | POU2F2 | PGS1 | RMDN3 | TXNDC17 |
| PTGES2 | DSP | DVL1P1 | UCK2 | BHLHE40 | COX16 | ERBB4 | PALB2 | IMMP2L | GUCY1B1 | TXNDC2 |
| NME9 | SERPINA1 | LOC106736470 | UBE2V1 | BST1 | H2AX | FGFR2 | PANK2 | LCN1 | COP1 | TXNDC4 |
| CASP8 | GATA4 | CDK4 | NAA10 | CDH23 | EDEM3 | EZH2 | IGFBP5 | TRAM1 | CHAC2 | TXNDC8 |
| CD40 | EEF2 | HDAC4 | TAB1 | METAP1 | EFS | BRAF | RUNX3 | TNFAIP2 | CHCHD1 | TXNIP |
| ENO1 | UGT1A1 | SLC9A1 | VTI1B | HLF | MRPL41 | MET | NME3 | UGT1A7 | HDDC2 | TXNL1 |
| ATP7A | KAT2B | ZAP70 | CAP1 | PSIP1 | VEGFD | CHEK2 | SLC5A3 | LDHAL6B | MTERF3 | TXNRD1 |
| IDO1 | FANCC | IL2RA | GART | SLC24A2 | MELTF | FLT4 | SLC7A1 | TTC19 | DNAJC30 | TXNRD2 |
| FOXO3 | CHAT | PPIB | ATG3 | LMF1 | SLIRP | CDK5 | SMAD7 | TACO1 | MRPL47 | TXNRD3 |
| SRXN1 | SLC1A1 | PTPRF | ARCN1 | KCNMB3 | TRA | TLR3 | HSD17B7 | JPH1 | MRPL54 | UCP1 |
| INS | GNAI1 | ACAT1 | FAM20C | TMPRSS11A | COX17P1 | ATR | HSD3B1 | REEP5 | MRPL55 | UCP2 |
| OGG1 | CBR1 | HCK | MAFA | AASDH | MTCO2P12 | ADA | PLK2 | TBL2 | MT4 | VCRL1 |
| MMP2 | FECH | F2 | ALDOC | ZACN | TRC-GCA24-1 | GRIN2A | SLC30A2 | RDH13 | DCAF5 | VCRL2 |
| HSPB1 | SCARB1 | ALDOA | AIMP2 | CCN1 | CDK6 | PCSK9 | SLC25A19 | RAB11FIP5 | PRSS35 | BCL2A1 |
| GRB2 | RHOB | AK2 | ERLIN1 | MIR328 | AKT3 | PRKCG | SLC25A24 | RAB4B | SERHL2 | BNIP3L |
| IL1A | DAXX | ABCA1 | FLOT1 | CYP19A1 | CDK2 | PRKACA | SLC12A4 | QRSL1 | IFNA7 | CAPN3 |
| CDC42 | SULT1E1 | COL2A1 | ACOT7 | OGDH | AXL | VDR | SLC22A4 | TMEM126A | HSDL1 | GIT1 |
| ENOX2 | IL5 | RRM2 | BRD7 | SIRT6 | HK1 | GNAS | SLC13A3 | TMEM126B | SLC25A51 | GPHN |
| PPARG | ARF4 | PLG | LYVE1 | RAF1 | HDAC2 | ESRRB | PTN | PI3 | NUDT8 | LGALS13 |
| SLC11A1 | LTF | RAB7A | CLTB | CFL1 | TGFBR2 | CCND2 | PIAS4 | OXSM | NUDT13 | MAGT1 |
| ARHGDIB | CRYZ | STK4 | CHMP4B | TAT | GCK | DRD2 | PSMD9 | TRMU | TOMM40L | MEIS1 |
| GLRX5 | SKP1 | KRT18 | MYL1 | TUBB | CACNA1H | CDH1 | PHOX2B | DNASE2 | TWNK | MIR6855 |
| TXNDC9 | RPL27 | CAMK2G | CDC37 | NLRP3 | CBL | GRIN2B | TRAF3IP2 | DUSP13 | UQCC1 | NEWENTRY |
| SH3BGRL3 | SSR4 | HADHB | GPLD1 | RPS6 | DNMT1 | CDH2 | LAP3 | NCOA4 | PTRH1 | SCRIB |
| TXNDC16 | LPA | ECHS1 | EIF3A | PRKN | SPARC | PRKCH | UGCG | NDUFA3 | TMBIM4 | SH3BGRL2 |
| BRCA1 | RPL12 | PAX6 | EHD1 | IL1R1 | TNFRSF10B | PFKM | TRIB3 | CNGA2 | TMEM65 | SNAI2 |
| FXN | RPL13A | KAT2A | EIF1AX | AOX1 | CASP2 | NR5A1 | TRPM1 | SEC14L2 | TMEM45B | STEAP3 |
| CRAT | MYO1D | YWHAQ | PFN2 | S100A9 | MAP2K3 | NGF | TAC1 | NDUFB7 | TMEM186 | TTN-AS1 |
| PDR | PLXNC1 | ACO2 | PRPF6 | RPL27A | GRIN1 | SMARCA4 | SYP | MT1E | IFNA17 | USP17L9P |
| CCND1 | RCC2 | DLAT | NPHS2 | CYB561 | RXRA | RARB | RETN | MRPL1 | NAF1 | WNT5A |
| MAPK7 | LIMA1 | MYOD1 | PABPC4 | SESN2 | SLC6A3 | TBXAS1 | TCL1A | RAB3D | HIGD2A |  |
| PYCR1 | MYO1B | CDC25B | PAFAH1B3 | PRKAG1 | TTR | TEK | TARS2 | IFI30 | CACUL1 |  |
| MAOB | NOC2L | CD79A | SNX1 | PHB | TUBB3 | STAT6 | ING1 | ISCA2 | MIEF1 |  |
| DYNLL1 | INTS7 | PSAP | SAR1A | MAP3K14 | LDLR | ROCK1 | P2RX2 | THEM4 | METTL17 |  |
| MAPK3 | TMEM37 | PCK2 | RPN2 | HP | LIMK1 | VLDLR | PDYN | TIMELESS | FSIP2 |  |
